# *MMV_Im2Im*: an open-source microscopy machine vision toolbox for image-to-image transformation

**DOI:** 10.1093/gigascience/giad120

**Published:** 2024-01-27

**Authors:** Justin Sonneck, Yu Zhou, Jianxu Chen

**Affiliations:** Leibniz-Institut für Analytische Wissenschaften – ISAS – e.V., Bunsen-Kirchhoff-Str. 11, Dortmund 44139, Germany; Faculty of Computer Science, Ruhr-University Bochum, Universitätsstraße 150, Bochum 44801, Germany; Leibniz-Institut für Analytische Wissenschaften – ISAS – e.V., Bunsen-Kirchhoff-Str. 11, Dortmund 44139, Germany; Leibniz-Institut für Analytische Wissenschaften – ISAS – e.V., Bunsen-Kirchhoff-Str. 11, Dortmund 44139, Germany

**Keywords:** deep learning, microscopy image analysis, open-source

## Abstract

Over the past decade, deep learning (DL) research in computer vision has been growing rapidly, with many advances in DL-based image analysis methods for biomedical problems. In this work, we introduce *MMV_Im2Im*, a new open-source Python package for image-to-image transformation in bioimaging applications. *MMV_Im2Im* is designed with a generic image-to-image transformation framework that can be used for a wide range of tasks, including semantic segmentation, instance segmentation, image restoration, image generation, and so on. Our implementation takes advantage of state-of-the-art machine learning engineering techniques, allowing researchers to focus on their research without worrying about engineering details. We demonstrate the effectiveness of *MMV_Im2Im* on more than 10 different biomedical problems, showcasing its general potentials and applicabilities. For computational biomedical researchers, *MMV_Im2Im* provides a starting point for developing new biomedical image analysis or machine learning algorithms, where they can either reuse the code in this package or fork and extend this package to facilitate the development of new methods. Experimental biomedical researchers can benefit from this work by gaining a comprehensive view of the image-to-image transformation concept through diversified examples and use cases. We hope this work can give the community inspirations on how DL-based image-to-image transformation can be integrated into the assay development process, enabling new biomedical studies that cannot be done only with traditional experimental assays. To help researchers get started, we have provided source code, documentation, and tutorials for *MMV_Im2Im* at [https://github.com/MMV-Lab/mmv_im2im] under MIT license.

## Introduction

**Figure 1:**
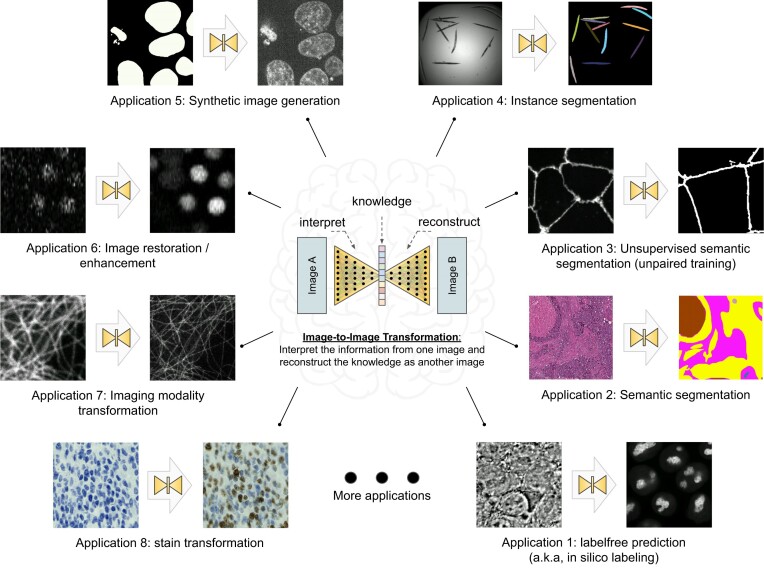
Overview of the image-to-image transformation concept and its example applications.

With the rapid advancements in the fields of machine learning (ML) and computer vision, computers can now transform images into new forms, enabling better visualization [1], better animation [2], and better information extraction [3] with unprecedented and continuously growing accuracy and efficiency compared to conventional digital image processing. These techniques have recently been adapted for bioimaging applications and have revolutionized image-based biomedical research [4–7]. In principle, these techniques and applications can be formulated as a general image-to-image transformation problem, as depicted in the central panel in Fig. 1. Deep neural networks are trained to perceive the information from the source image(s) and reconstruct the learned knowledge from source images(s) in the form of a new image(s) of the target type. The source and target images can be real or simulated microscopy images, segmentation masks, or their combinations, as exemplified in Fig. 1. Since these underlying methods share the same essential spirit, a natural question arises: is it possible to develop a single generic codebase for deep learning (DL)–based image-to-image transformation applicable to various biomedical studies?

In this article, we introduce *MMV_Im2Im*, an open-source microscopy machine vision (MMV) toolbox for image-to-image transformation, and demonstrate its applications in over 10 biomedical tasks of various types by performing more than 30 experiments. Currently, *MMV_Im2Im* supports handling 2D to 5D microscopy images for supervised image-to-image translation (e.g., label-free determination [4], imaging modality transformation [5, 8]), supervised image restoration [6], supervised semantic segmentation [9], supervised instance segmentation [10, 11], unsupervised semantic segmentation [12], and unsupervised image-to-image translation and synthetization [13]. The toolbox will continuously grow with more and more methods, such as self-supervised learning-based methods, ideally also with contributions from the open-source community.

Why do we need such a single generic codebase for all DL-based microscopy image-to-image transformation? *MMV_Im2Im* is not simply a collection of many existing methods but rather has a systematic design for generality, flexibility, simplicity, and reusability, attempting to address several fundamental bottlenecks for image-to-image transformation in biomedical applications, as highlighted below.

### Feature 1: universal boilerplate with state-of-the-art ML engineering

#### Bottleneck: existing code not easy to understand or to extend or reuse

Our package *MMV_Im2Im* employs pytorch-lightning [14] as the core in the backend, which offers numerous benefits, such as readability, flexibility, simplicity, and reusability. First of all, have you ever had the moment when you wanted to extend someone’s open-source code to suit your special ML needs but found it so difficult to figure out where and how to extend, especially for complex methods? Or, have you ever encountered the situation where you want to compare the methods and code from 2 different papers, even solving the same problem (e.g., semantic segmentation), but not quite easy to grasp quickly since the 2 repositories are implemented in very different ways? It is not rare that even different researchers from the same group may implement similar methods in very different manners. This is not only a barrier for other people to learn and reuse the open-source code, but it also poses challenges for developers in maintenance, further development, and interoperability among different packages. We follow the pytorch-lightning framework and carefully design a universal boilerplate for image-to-image transformation for biomedical applications, where the implementation of all the methods shares the same modularized code structure. For all PyTorch users, this greatly lowers the learning curve for people to read and understand the code and makes implementing new methods or extending existing methods simple and fast, at least from an engineering perspective.

Moreover, as ML scientists, have you ever been overwhelmed by different training tricks for different methods or been curious about if certain state-of-the-art training methods can boost the performance of existing models? With the pytorch-lightning backend, *MMV_Im2Im* allows you to enjoy different state-of-the-art ML training engineering techniques without changing the code, for example, stochastic weight averaging [15], single precision training, automatic batch size determination, different optimizers, different learning rate schedulers, easy deployment on different devices, distributed training on multi-GPU (even multinode), logging with common loggers such as Tensorboard, and so on. In short, with the pytorch-lightning–based universal boilerplate, bioimaging researchers can really focus on research and develop novel methods for their biomedical applications, without worrying about the ML engineering works (which are usually lacking in non–computer science labs).

### Feature 2: modularization and human-readable configuration system

#### Bottleneck: dilemma between simplicity and flexibility

The toolbox is designed for people with or without extensive experience with ML and Python programming. It is not rare to find biomedical image analysis software that is very easy to use on a set of problems, but very hard to extend or adjust to other different but essentially related problems, or find some with great flexibility with tunable knobs at all levels, but unfortunately not easy for inexperienced users. To address this issue, we designed the toolbox in a systematically modularized way with various levels of configurability. One can use the toolbox with a single command as simple as run_im2im –config train_semanticseg_3d –data.data_path/path/to/data or make customization on details directly from a human-readable configuration file, such as choosing batch normalization or instance normalization in certain layers of the model, or adding extra data augmentation steps, and so on. We provide an extensive list of more than 20 example configurations for various applications and comprehensive documentation to address common questions for users as reference. For users preferring graphical interface, another napari plugin for the MMV toolbox has been planned as the extension of *MMV_Im2Im* (see Discussion for details).

In addition, the modularization and configuration system is designed to allow not only configuring with the elements offered by the package itself but also any compatible elements from a third-party package or from a public repository on GitHub. For example, one can easily switch the 3D neural network in the original *Embedseg* method to any customized U-Net from FastAI by specifying the network as fastai.vision.models.unet. Such painless extendability releases the power of the toolbox, amplifies the benefit of the open-source ML community, and upholds our philosophy of open science.

### Feature 3: customization for biomedical imaging applications

#### Bottleneck: not enough consideration for specific challenges in microscopy images in general DL toolboxes

The original idea of a general toolbox actually stemmed from the OpenMMLab project [16], which provides generic codebases for a wide range of computer vision research topics. For instance, *MMSegmentation* [17] is an open-source toolbox for semantic segmentation, supporting unified benchmarking and state-of-the-art models ready to use out-of-box. It has become one of most widely used codebases for research in semantic segmentation (2.3 K forks and 6.5 K stars on GitHub as of 29 September 2023). This inspires us to develop *MMV_Im2Im* to facilitate research in image-to-image transformation with a special focus on biomedical applications.

First of all, different from general computer vision datasets, such as ImageNet [18], where the images are usually small 2D RGB images (e.g., 3 × 256 × 256 pixels), biomedical applications usually involve large-scale high-dimensional data (e.g., 500 images of 4 × 128 × 2,048 × 2,048 voxels). To deal with this issue, we employ the PersistentDataset in MONAI [19] with partial loading and sampling support, as well as delayed image reading powered by aicsimageio [20] as default (configurable if another dataloader is preferred). As a result, in our stress test, training a 3D nuclei instance segmentation model with more than 125,000 3D images can be conducted efficiently on a day, even with limited resources.

Second, because microscopy data are not restricted to 2D, we reimplement common frameworks, such as fully convolutional networks (FCNs), conditional generative models, cycle-consistent generative models, and so on, in a generic way to easily switch between different dimensionalities for training. During inference, up to 5D images (channel × time × Z × Y × X) can be directly loaded as the input without presplitting into smaller 2D/3D chunks.

Third, the toolbox prepacks common functionalities specific to microscopy images. For example, we incorporate the special image normalization method introduced in [4], where only the middle chunk along the Z dimension of 3D microscopy images will be used for calculating the mean and standard deviation of image intensity for standard normalization. Also, 3D light microscopy images are usually anisotropic (i.e., much lower resolution along Z than XY dimension). So, we adopt the anisotropic variation of UNet as proposed in [21].

Finally, to deploy the model in production, a model trained on small 3D patches sometimes needs to be applied not only on much larger images. Combining the efficient data handling of aicsimageio [20] and the sliding window inference with Gaussian weighted blending, the toolbox can yield efficient inference without visible stitching artifacts in production.

All in all, the *MMV_Im2Im* toolbox stands on the shoulders of many giants in the open-source software and ML engineering communities (pytorch-lightning, MONAI, aicsimageio, etc.) and is systematically designed for image-to-image transformation R&D for biomedical applications. The source code of *MMV_Im2Im* is available at [22]. This manuscript is generated with the open-source package Manubot [23]. The manuscript source code is available at [24].

## Results

In this section, we showcase the versatility of the *MMV_Im2Im* toolbox by presenting over 10 different biomedical applications across various R&D use cases and scales. All experiments and results in this section were conducted on publicly available datasets released with other publications and our scripts (for pulling the public dataset online and data wrangling) and configuration files (for setting up training and inference details), both included in the *MMV_Im2Im* package. Our aim is to make it easy to reproduce all of the results in this article and, more important, use these data and scripts to get familiar with the package and adapt to new problems of users’ interest. It is important to note that the aim of these experiments was not to achieve the best performance on each individual task, as this may require further hyperparameter tuning (see Discussion section for more details). Rather, the experiments were intended to demonstrate the package’s different features and general applicability, providing a holistic view of image-to-image transformation concepts to biomedical researchers. We hope that these concepts will help researchers integrate AI into traditional assay development strategies and inspire computational and experimental codesign methods, enabling new biomedical studies that were previously unfeasible.

### Label-free prediction of nuclear structure from 2D/3D brightfield images

The label-free method refers to a DL method that can predict fluorescent images directly from transmitted light brightfield images [4]. Compared to brightfield images, fluorescent images can resolve subcellular structures in living cells at high resolution but with the cost of expensive and slow procedures and high phototoxicity. The label-free method provides a new perspective in assay development to conduct integrated computational analysis of multiple organelles only with a single brightfield image acquisition. In our first demonstration, we applied *MMV_Im2Im* to build 2D/3D models that can predict fluorescent images of nuclear structures from brightfield images. For 3D models, we also compared (i) different image normalization methods, (ii) different network backbones, and (iii) different types of models.

It should be noted that while we recognize the importance of systematically evaluating the predictions, such an analysis falls outside the scope of this article. We argue that an appropriate evaluation methodology should depend on specific downstream quantitative analysis goals (e.g., [25–27]). For example, if our aim is to quantify the size of nucleoli, we must compare the segmentation derived from real nucleoli signals to that of the predicted nucleoli segmentation, ensuring that measurements from both are consistent. Alternatively, if the goal is to localize the nucleoli roughly within the cell, Pearson correlation may be a more appropriate metric. In this work, we concentrate on visual inspection, using Pearson correlation and structural similarity as a rough quantitative reference. Our intent is to demonstrate the utility of our *MMV_Im2Im* package and leave appropriate evaluations to users in their specific problems in real studies.

#### 2D label-free

**Figure 2:**
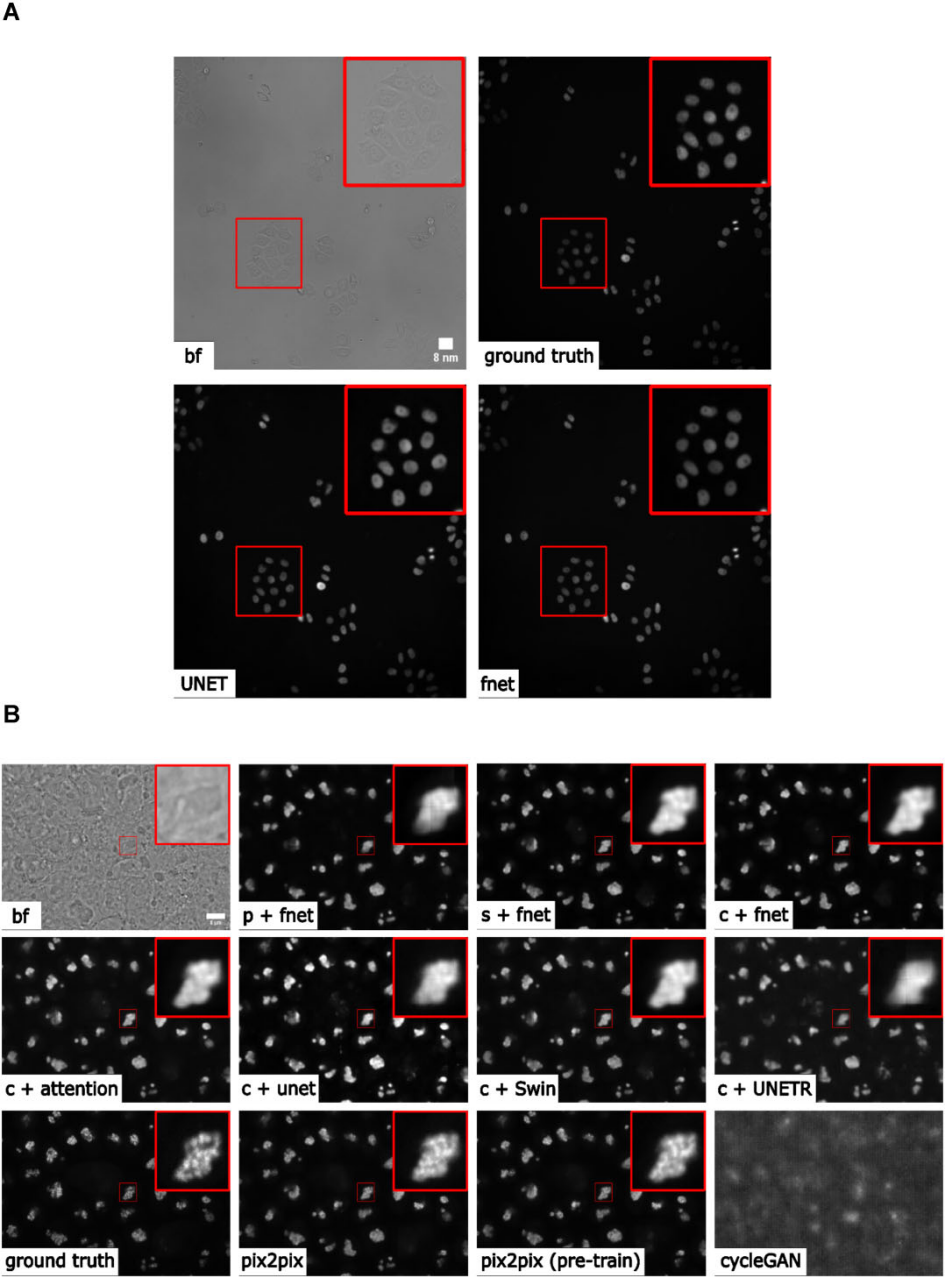
(A) Example of 2D label-free results. (B) Overview of various 3D label-free results obtained by different training strategies. p/c/s refers to percentile normalization, center normalization, and standard normalization, respectively (see main text for details). (The contrast of grayscale images was adjusted using ImageJ’s autoscale.)

We started with a simple problem using 2D images from the HeLa “Kyoto” cells dataset [28]. For all images, we took the brightfield channel and the mCherry-H2B channel out of the multichannel time-lapse movies. The 2D images were acquired at 20× with 0.8 NA and then downscaled by 4 (pixel size: 0.299 × 0.299 nm). Example predictions can be found in Fig. 2A. We compared a basic UNet model [9] and a 2D version of the fnet model in [4]. The fnet model achieved slightly more accurate predictions than the basic UNet, as seen in Fig. 2A.

#### 3D label-free

We tested with 3D images from the human induced pluripotent stem cell (hiPSC) single-cell image dataset [29]. Specifically, we extracted the brightfield channel and the structure channel from the full field-of-view (FOV) multichannel images from the HIST1H2BJ, FBL, NPM1, and LMNB1 cell lines, so as to predict from one brightfield image various nuclear structures, histones, nucleoli (dense fibrillar component via fibrillarin), nucleoli (granular component via nucleophosmin), and nuclear envelope, respectively. Images were acquired at 100× with 1.25 NA (voxel size: 0.108 × 0.108 × 0.29 microns).

**Figure 3:**
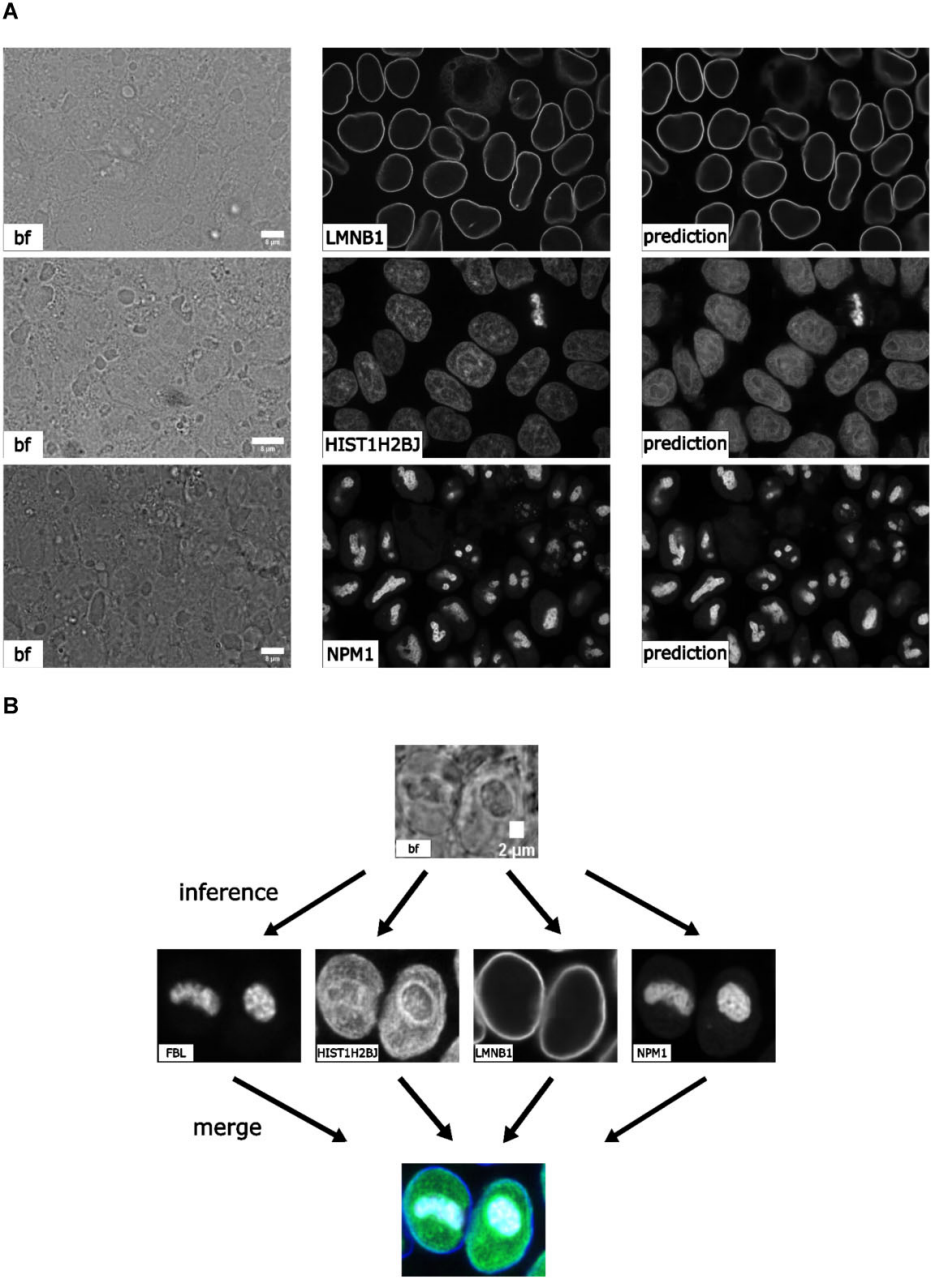
(A) Comparison of predictions of different 3D label-free models and ground truth. (B) Predictions of the different label-free models using the same brightfield image as input, which provides a deep insight into the nuclear structure. This would not be possible with brightfield imaging alone and is enabled by the application of the label-free approach. (The contrast of grayscale images was adjusted using ImageJ’s autoscale.)

We conducted 3 groups of comparisons (see results in Fig. 2B). First, we compared 3 different image normalization methods for 3D images: percentile normalization, standard normalization, and center normalization [4]. Percentile normalization refers to cutting the intensity out of the range of [0.5, 99.5] percentile of the image intensity and then rescaling the values to the range of [−1, 1], while the standard normalization is simply subtracting mean intensity and then dividing by the standard deviation of all pixel intensities. Center normalization is similar to standard normalization, but the statistics are calculated only around the center along the Z-axis [4]. One could easily test different percentiles or rescaling to [0, 1] instead of [−1, 1]. Qualitatively, we found center normalization slightly more accurate and more robust than the other two (see first row of Fig. 3B).

Second, we compared different network backbone architectures, including the original fnet model [4], an enhanced UNet [30], the attention UNet [31], and 2 transformer-based models, SwinUNETR [32] and UNETR [33] (all with center normalization). Inspecting the predictions on a holdout validation set suggested that fnet achieved the best performance.

Finally, we showed the comparison between 3 different types of models, an FCN-type model (i.e., fnet), a pix2pix-type model, and a CycleGAN-type model. For fair comparison, we used fnet as the same backbone for all 3 types of models. In theory, the pix2pix-type model can be trained in 2 different ways: from scratch or initializing the generator with a pretrained fnet (trained as FCN). Examples of the comparison results are shown in the last 2 rows in Fig. 3B. Visually, it is evident that the additional adversarial components (i.e., the discriminator) could generate images with a more realistic appearance than a typical FCN-type model alone, but again, we leave the appropriate quantitative evaluations to users’ specific biomedical studies.

From the experiments above, we found that center normalization + pix2pix with fnet as the generator achieved the best overall performance qualitatively. So, we employed the same strategy on all other nuclear structures. At the end, we had 4 different label-free models, each predicting one different nuclear structure from 3D brightfield images. As an example of evaluation, we calculated the Pearson correlation, the structural similarity, and the peak signal-to-noise ratio on holdout validation sets. The results are summarized in Table 1. Again, these numbers were merely examples of evaluation; systematic evaluation based on each specific biological problem would be necessary before deployment. Figure 3A shows the comparison of each predicted structure and its ground truth, while Fig. 3B shows one example of all 4 different structures predicted from a single unseen brightfield image. This would permit an integrated analysis of 4 different nuclear components that could hardly be acquired simultaneously in real experiments and real images.

**Table 1:** Evaluation of the final 3D label-free models for 4 different nuclear structures

Dataset	Pearson correlation	Structural similarity	Peak signal-to-noise ratio
FBL	0.902 ± 0.014	0.864 ± 0.029	33.559 ± 1.182
HIST1H2BJ	0.880 ± 0.022	0.735 ± 0.070	27.307 ± 2.832
LMNB1	0.883 ± 0.020	0.703 ± 0.060	29.582 ± 1.793
NPM1	0.939 ± 0.009	0.846 ± 0.027	32.636 ± 1.040

### 2D semantic segmentation of tissues from H&E images

**Figure 4:**
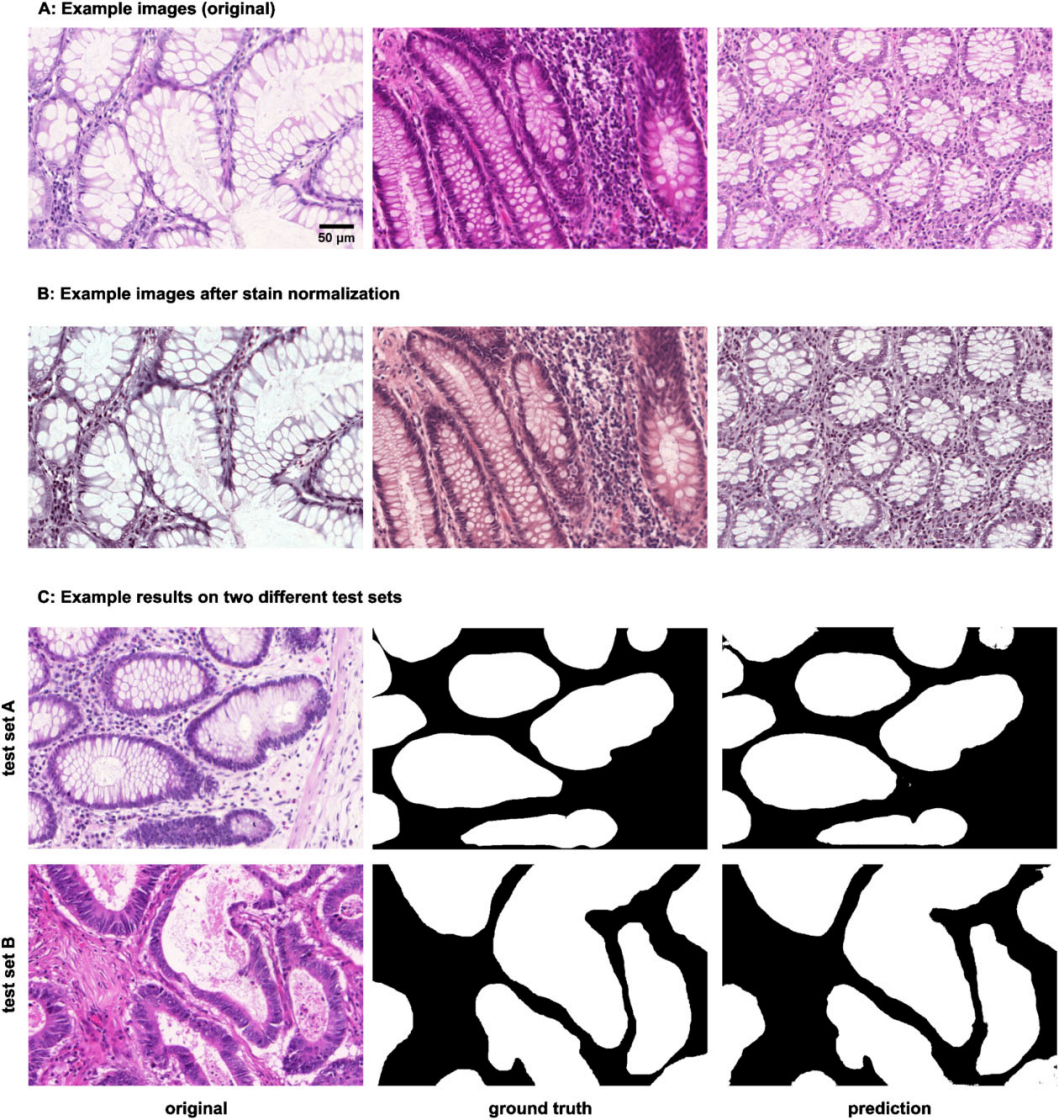
Example results of 2D semantic segmentation of a gland in H&E images. A and B provide insight into the stain normalization implemented in *MMV_Im2Im*. C compares a raw example image before stain normalization and prediction to the ground truth for each test set.

Segmentation is a common image-processing task and can be considered a special type of image-to-image transformation, where the generated images are segmentation masks. DL-based methods have achieved huge success in semantic segmentation in biomedical images. In this example, we demonstrated *MMV_Im2Im* on a pathology application to segment glands from hematoxylin and eosin (H&E)–stained tissue images from the 2015 Gland Segmentation challenge [34, 35]. Stain normalization is an important preprocessing step in order to develop models robust to stain variation and tissue variations. *MMV_Im2Im* included a classic stain normalization method [36] as a preprocessing step. The effect of stain normalization can be observed in Fig. 4A, B. We trained a simple attention UNet model [31]. Evaluated on the 2 different holdout test sets, the model achieved an F1-score of 0.904 ± 0.060 and 0.861 ± 0.117 on test set A and test set B, respectively. The performance was competitive compared to the methods reported in the challenge report [35], especially with much more consistent performance across the 2 different test sets. Example results can be found in Fig. 4C.

### Instance segmentation in microscopy images

Instance segmentation is a type of segmentation problem that goes beyond semantic segmentation. The goal is to differentiate not only between different types of objects but also different instances of the same type of objects. Currently, the *MMV_Im2Im* package supports EmbedSeg-type models. The major benefit of EmbedSeg-type models is their agnosticism to the morphology and dimensionality of the object instances, compared to other models such as StarDist [37, 38], SplineDist [39], and Cellpose [40]. For example, different from the others, EmbedSeg-type models are even able to generate instance segmentation where each instance contains multiple connected components. Additional frameworks such as Omnipose [41] will be supported in future versions. Another mainstream category of instance segmentation methods are detection-based models, such as Mask-RCNN [42]. However, these models are better suited to the detection framework rather than image-to-image transformation (see Discussion section for details).

The *EmbedSeg*-type models were reimplemented according to the original study [10, 11] following the generic boilerplate in *MMV_Im2Im*, with significant improvement. First of all, following the modular design of *MMV_Im2Im*, it is flexible to use different neural network models as the backbone. For 3D anisotropic microscopy images, the original backbone ERFNet [43] does not take the anisotropic dimensions into account and therefore may not perform well or even be applicable. In this scenario, it is straightforward to employ another anisotropic neural network bone, such as the anisotropic U-Net in [21] or the anisotropic version of Dynamic U-Net in MONAI. Second, we significantly improved the training strategy. The original version requires precropping patches centered on each instance and precalculating the center images and class images. This may generate a massive amount of additional data on the disk. More important, such precropping makes data augmentation nearly impossible, except the simple ones like flipping (otherwise, the precalculated centers might be wrong), and also greatly undersamples around negative cases (e.g., background). For example, we have observed that for an EmbedSeg model training only with patches centered on instances, the model may suffer from degraded performance during inference when there are a large amount of background areas without any instances. Again, following the modular design of *MMV_Im2Im*, it is now possible to do on-the-fly data augmentation and patch sampling, even weighted patch sampling. Third, our improved *EmbedSeg*-type models can accept an exclusion mask so that certain parts of the images can be ignored during training. This is especially useful for partially annotated ground truth. For large images, it could be extremely time-consuming to require every single instance to be annotated. The exclusion masks can address this bottleneck. Another extension compared to the original implementation was that the *MMV_Im2Im* package made sliding windowing inference straightforward and therefore permitted easy handling of images of any size during inference.

In this work, we tested on both 2D and 3D instance segmentation problems. Going from 2D to 3D is not a simple generalization from 2D models by switching 2D operations with 3D operations, but with many practical challenges. Large GPU footprint is one of the biggest issues, which makes many training strategies common in 2D not feasible in 3D (e.g., limited mini-batch size). *MMV_Im2Im* is able to take advantage of state-of-the-art ML engineering methods to efficiently handle 3D problems. For example, by using effective half-precision training, one can greatly reduce GPU memory workload for each sample and therefore increase the batch size or the patch size. When multiple GPUs are available, it is also possible to easily take advantage of the additional resources to scale up the training to multiple GPU cards, even multiple GPU nodes. As a demonstration, we applied *EmbedSeg*-like models to a 2D problem of segmenting *Caenorhabditis elegans* from widefield images [44], as well as a 3D problem of nuclear segmentation from fluorescent and brightfield images from the hiPSC single-cell image dataset [29].

**Figure 5:**
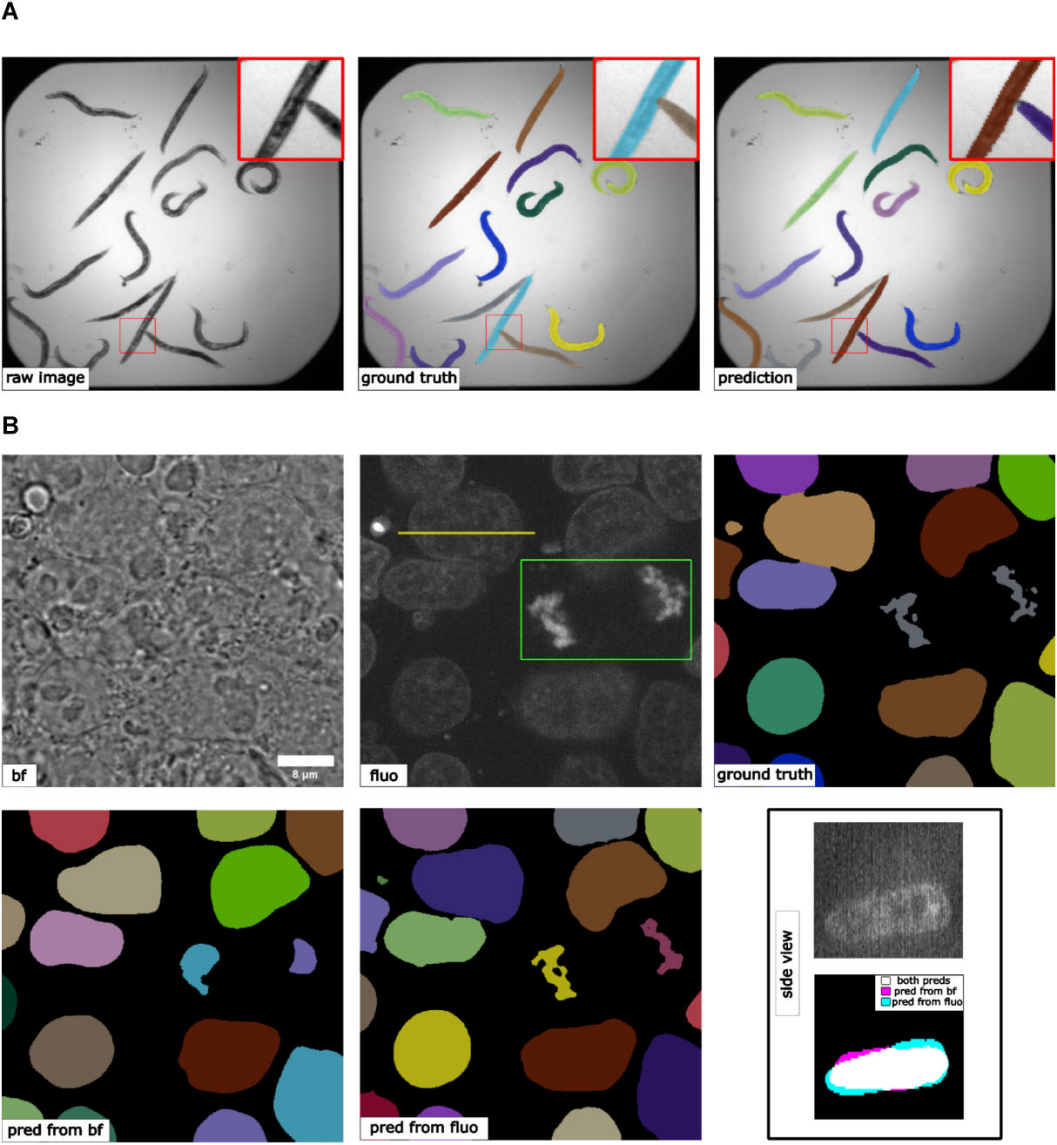
(A) Results of 2D instance segmentation of *C. elegans*. A minor error can be observed in the zoom-in window. (B) Results of 3D nuclear instance segmentation from fluorescent images and brightfield images. The green box in the fluorescent image highlights a mitotic example. The side view panel shows the segmentation of one specific nucleus along the line annotated in the fluorescent image from the side. The contrast of grayscale images was adjusted using ImageJ’s autoscale.

For the 2D problem, we adopted the same network backbone as in the original *EmbedSeg* paper. Example results on a small holdout set of 5 images are shown in Fig. 5A (average precision at 50 = 0.866 ± 0.163), which is comparable to the original published results [11]. For the 3D problem, the original backbone is not directly applicable, due to the before mentioned anisotropic issue, and the images in the dataset do not contain enough Z-slices to run through all down-sampling blocks in 3D. The anisotropic UNet [21] is used here. The segmentation results obtained from the public dataset [29] contain nuclear instance segmentation of all cells. But, the cells touching the image borders are ignored from downstream analysis [29] and therefore not curated. In other words, the segmentation from this public dataset can only be used as high-quality nuclear instance segmentation ground truth after excluding the areas covered by cells touching the image borders [29]. Therefore, the exclusion masking function in *MMV_Im2Im* is very helpful in this example.

Example results are presented in Fig. 5B. The green box highlighted a mitotic cell (the DNA signals forming “spaghetti” shapes). The average precision at 50 for the fluorescence model is 0.827 ± 0.082, and it can be seen that the fluorescence model is able to distinguish the complex DNA signal from the background. Even holes can appear in the predicted segmentation, allowing the prediction of very complex shapes that are theoretically not feasible for other instance segmentation models like StarDist or Cellpose. Additionally, *EmbedSeg*-type models are able to assign spatially unrelated structures to the same instance (see Fig. 5, bottom). Nuclear instance segmentation from brightfield images was much more challenging than from fluorescent images (average precision at 50 = 0.622 ± 0.101).

### Comparing semantic segmentation and instance segmentation of organelles from 3D confocal microscopy images

**Figure 6:**
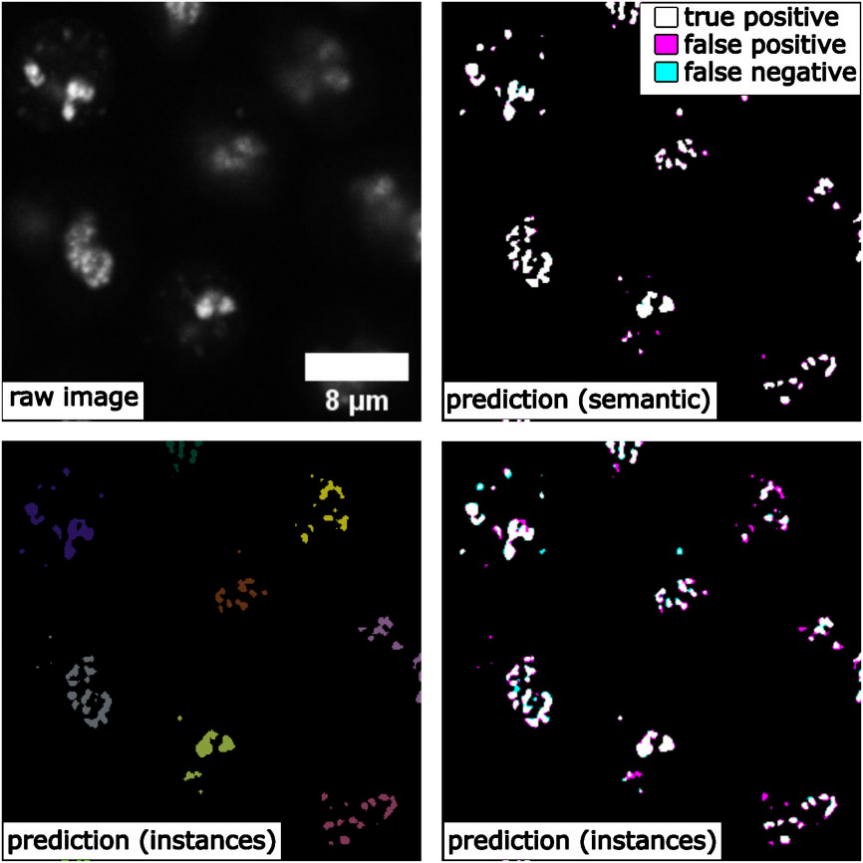
Comparing 3D semantic segmentation and 3D instance segmentation results on confocal microscopy images of fibrillarin (showing a middle Z-slice of a 3D stack), showing true-positive, false-negative, and false-positive pixels.

We did a special comparison in this subsection to further illustrate the difference between semantic and instance segmentations. We took the 3D fibrillarin dataset from [29]. There are multiple channels in each 3D image, including DNA dye, membrane dye, and the structure channel (i.e., fibrillarin in this case). The original fibrillarin segmentation released with the dataset is a semantic segmentation (0 = background, 1 = fibrillarin). With the additional cell segmentation available in the dataset, we can know which groups of segmented fibrillarin belong to the same cell. Then, we can convert the original 3D fibrillarin semantic segmentation ground truth into 3D instance segmentation ground truth (fibrillarin pixels belonging to the same cell are grouped as a unique instance). Sample images and results are shown in Fig. 6. We can observe that the semantic segmentation model is able to achieve good accuracy in determining pixels from the fibrillarin signals (F1 = 0.958 ± 0.008). Meanwhile, the instance segmentation can group them properly (average precision at 50 = 0.795 ± 0.055) so that fibrillarin masks from the same cell are successfully identified as unique instances, even without referring to the cell membrane channel or cell segmentation results. This is not a simple grouping step based on distance, since the fibrillarin signals from tightly touching nuclei may exist very close to each other.

### Unsupervised semantic segmentation of intracellular structures from 2D/3D confocal microscopy images

Large amounts of high-quality segmentation ground truth are not always available or may require endless effort to collect for a segmentation task. CycleGAN-based methods have opened up a new avenue for segmentation without the need for pixel-wise ground truth [12]. In this subsection, we demonstrate an unsupervised learning-based segmentation method on 4 examples: 2D tight-junction (via ZO1) segmentation from 2D FP-tagged ZO1 images (max-projected from 3D stacks) and segmentation of nuclei, mitochondria, and Golgi from 3D confocal microscopy images.

**Figure 7:**
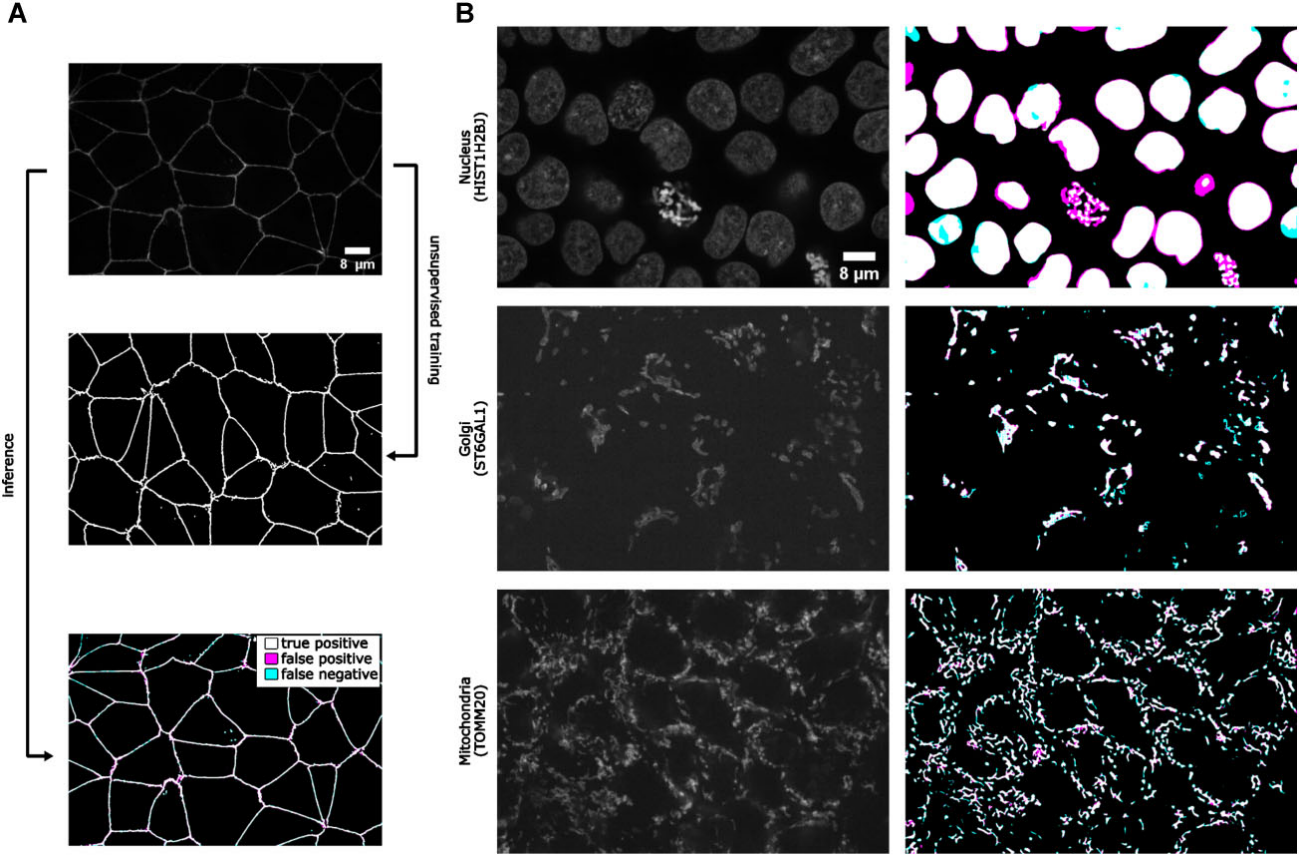
(A) Illustration of the unsupervised learning scheme and results in the 2D tight-junction segmentation problem. (B) Example 3D segmentation results (only showing a middle Z-slice) from models obtained by unsupervised learning. The contrast of grayscale images was adjusted using ImageJ’s autoscale.

To perform unsupervised learning, we used raw images from the hiPSC single-cell image dataset [29], as well as their corresponding segmentations (may not be absolute pixel-wise ground truth but have gone through systematic evaluation to ensure the overall quality). We shuffled the raw images and their segmentations to generate a set of simulated segmentation masks. A demonstration of the concept is illustrated in Fig. 7A. Example results for all 3D models are shown in Fig. 7B, and the F1-scores on the test set are summarized in Table 2.

**Table 2:** F1-scores of the unsupervised semantic segmentation predictions

Dimensionality	Dataset	F1-score	# of test data
2D	Tight junction	0.906 ± 0.011	18
3D	Nucleus	0.836 ± 0.081	31
3D	Golgi	0.689 ± 0.057	44
3D	Mitochondria	0.804 ± 0.015	54

For the 2D example, we saw that the unsupervised training provides a valuable segmentation, which is reflected by the F1-score in Table 2. For the 3D examples, it has been suggested that the quality of unsupervised nuclei segmentation could be further improved with additional simulation strategies [12]. Overall, we believe that unsupervised learning offers an effective way to generate preliminary segmentation, which can be further refined through active learning such as the iterative DL workflow described in [21].

### Generating synthetic microscopy images from binary masks

Generating a large amount of synthetic microscopy images can be an important step in developing image analysis methods. Synthetic images offer a way to train other DL models, such as self-supervised pretraining, using a diverse set of images without the need for large amounts of real-world data. As long as the synthetic images are generated with sufficient quality, it is possible to have an unlimited amount of training data for certain applications. Moreover, synthetic images can be used to evaluate other models when validation data are difficult to obtain. In this study, we demonstrate that *MMV_Im2Im* can generate 2D/3D synthetic microscopy images with high realism and validity, using a subset of data collected from the hiPSC single-cell image dataset [29], in either a supervised or an unsupervised manner.

**Figure 8:**
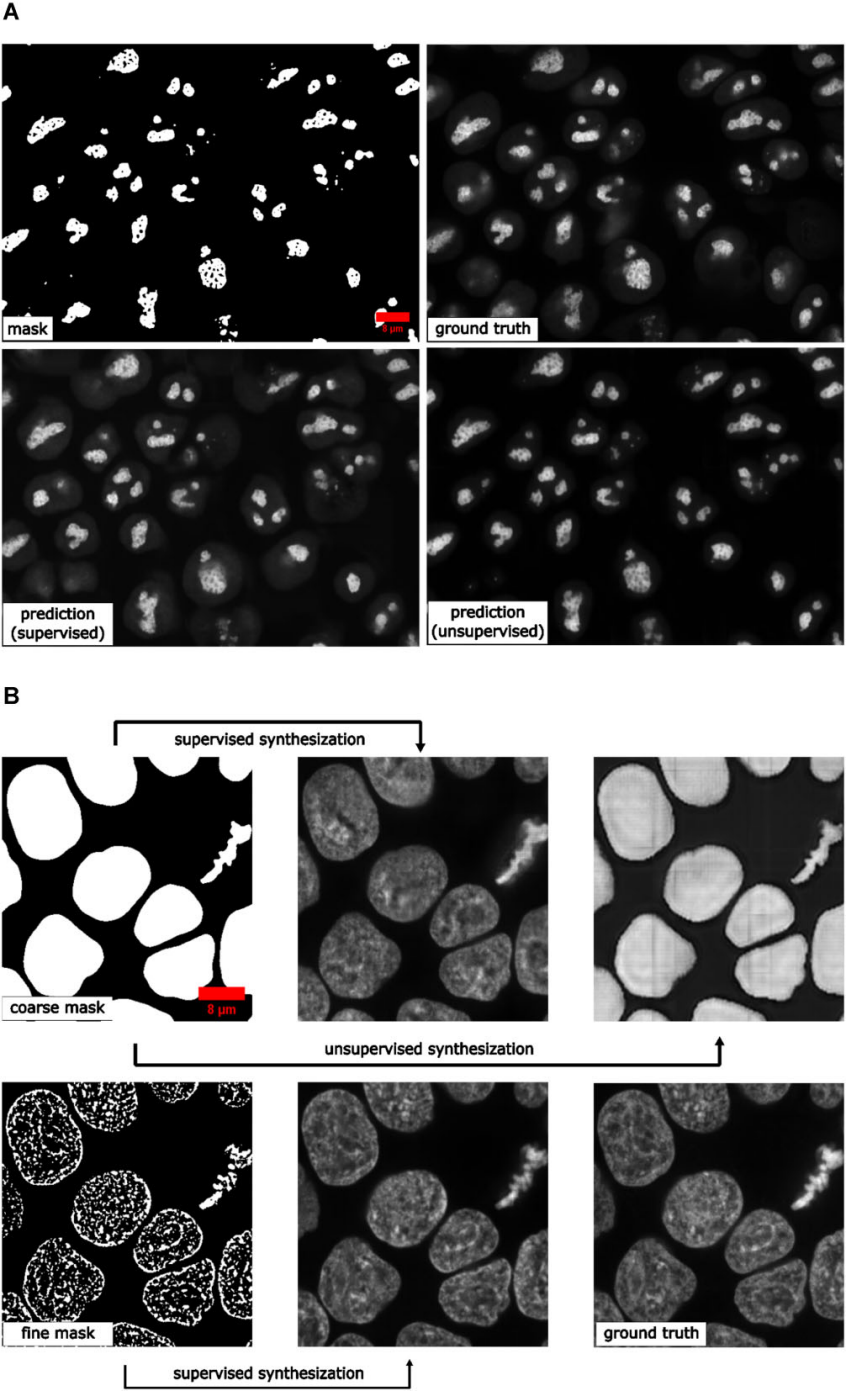
Example results of (A) 2D synthetic fluorescent images of nucleoli (via NPM1) and (B) 3D synthetic fluorescent images of H2B (middle Z-slices of a Z-stack) with a coarse mask and a fine mask as the input.

For 2D demonstration, we extracted the middle Z-slice from NPM1 images as the training target, while using the NPM1 segmentation results as the input binary masks. With the paired “mask + microscopy image” data, we could train the model in a supervised fashion or randomly shuffle the data to simulate the situation without paired data, which can be trained in an unsupervised fashion using the CycleGAN-type framework implemented in *MMV_Im2Im*. Example results can be found in Fig. 8A and Table 3. In general, the supervised synthesization can generate more realistic images than the unsupervised model.

**Table 3:** Results of the synthetic generation of microscopy images from binary masks

Dimensionality	Dataset	Training	Pearson correlation
2D	NPM1	Supervised	0.925 ± 0.019
2D	NPM1	Unsupervised	0.913 ± 0.023
3D	H2B_coarse	Supervised	0.841 ± 0.023
3D	H2B_coarse	Unsupervised	0.796 ± 0.035
3D	H2B_fine	Supervised	0.939 ± 0.009

For 3D demonstration, we use 3D H2B images with 2 different types of input masks. First, we attempted to generate synthetic images from a coarse mask (i.e., only the overall shape of the nucleus, available as nuclear segmentation from the dataset) with both supervised training and unsupervised training. The unsupervised model in *MMV_Im2Im* uses the CycleGAN-based approaches. So, the unsupervised training is actually already done within the unsupervised segmentation experiments. In other words, the unsupervised model works in a bidirectional way, from real microscopy images to binary masks, and also from binary masks to simulated microscopy images. Here, we could also do the inference in a different direction (from binary to simulated microscopy) using the model trained in the unsupervised segmentation section. The results are shown in Fig. 8B (row 1). The unsupervised synthesization can mostly “paint” the mask with homogeneous grayscale intensity, while the supervised model can simulate the textures to some extent. For a relatively large mask, it could be challenging for a model to fill in sufficient details to simulate real microscopy images (might be improved with diffusion-based models; see Discussion section).

We made another attempt with 3D masks containing finer details beyond the overall shapes. So, we employed the H2B structure segmentation results from the dataset (capturing the detailed nuclear components marked by histone H2B) as the input for supervised synthesization. The result is shown in Fig. 8B (row 2). Compared to the synthesization with coarse masks, the images simulated from fine masks exhibit a much more realistic appearance. As we can see, it is important to design the solutions with proper data. Preliminary quantitative evaluations on all synthesization experiments are summarized in Table 3.

### Image denoising for microscopy images

*MMV_Im2Im* can also be used to computationally reduce image noise or restore the data from various sources of imaging artifacts, so as to increase the feasibility and efficiency in downstream analysis. In the current version of *MMV_Im2Im*, the restoration model can only be trained in a fully supervised manner. Therefore, aligned low-quality and high-quality images are required for supervision, even though such paired data can be partially simulated [6]. Other methods, such as unsupervised learning-based solutions [45], will be made available within *MMV_Im2Im* in future versions.

**Figure 9:**
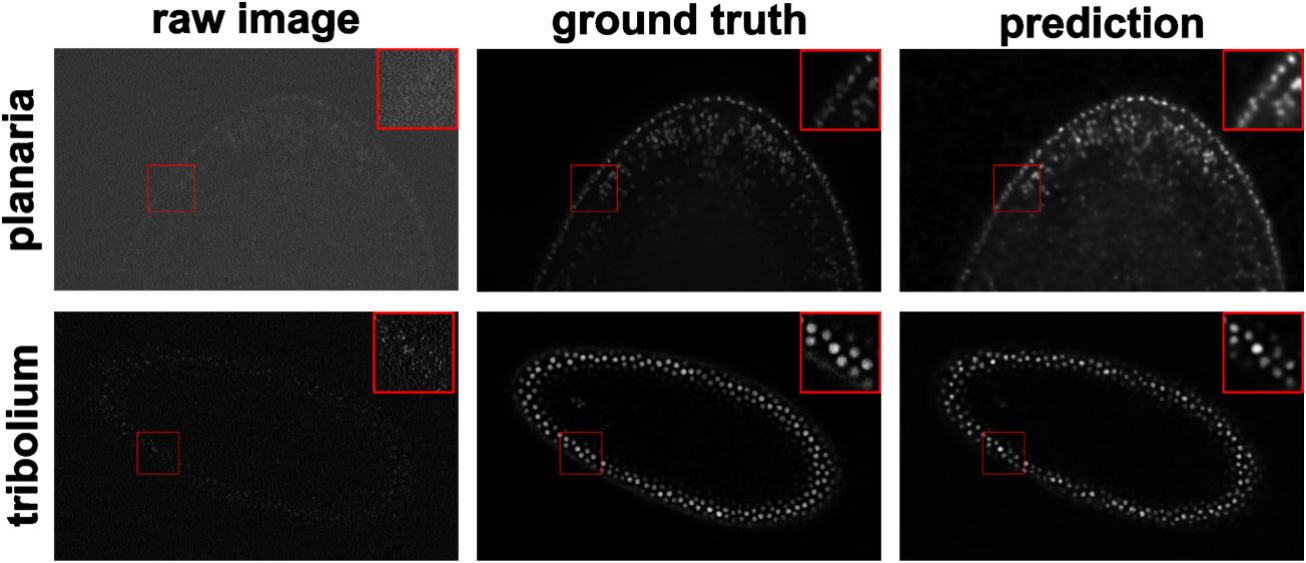
Denoising results of 3D images of nucleus-stained flatworm (planaria) and *Tribolium castaneum* embryos at a single Z-slice each. It can be seen that the predicted images have a greatly reduced SNR. Left: raw images (low SNR), middle: reference images (high SNR), right: predictions. The contrast of grayscale images was adjusted using ImageJ’s autoscale.

In this example, we presented an image denoising demonstration with sample data from [46]. The goal was to increase the quality of low signal-to-noise ratio (SNR) images of nucleus-stained flatworms (*Schmidtea mediterranea*, planaria) and lightsheet images of *Tribolium castaneum* (red flour beetle) embryos. The models were trained with paired data acquired with low and high laser intensity on fixed samples and then applied on live imaging data. For the nucleus-stained flatworm data (a test set of 20 images is available), the model achieved a Pearson correlation of 0.392 ± 0.065, while the Pearson correlation between the noisy raw and ground-truth images was 0.065 ± 0.057. For the red flour beetle dataset, the model has improved the Pearson correlation from 0.219 ± 0.045 to 0.444 ± 0.077 (6 images). Based on this and the results in Fig. 9, it can be observed that the low SNR images can be greatly improved. Systematic quantitative evaluations would be necessary to confirm the biological validity but beyond the scope of this article.

### Imaging modality transformation from 3D confocal microscopy images to stimulated emission depletion microscopy images

**Figure 10:**
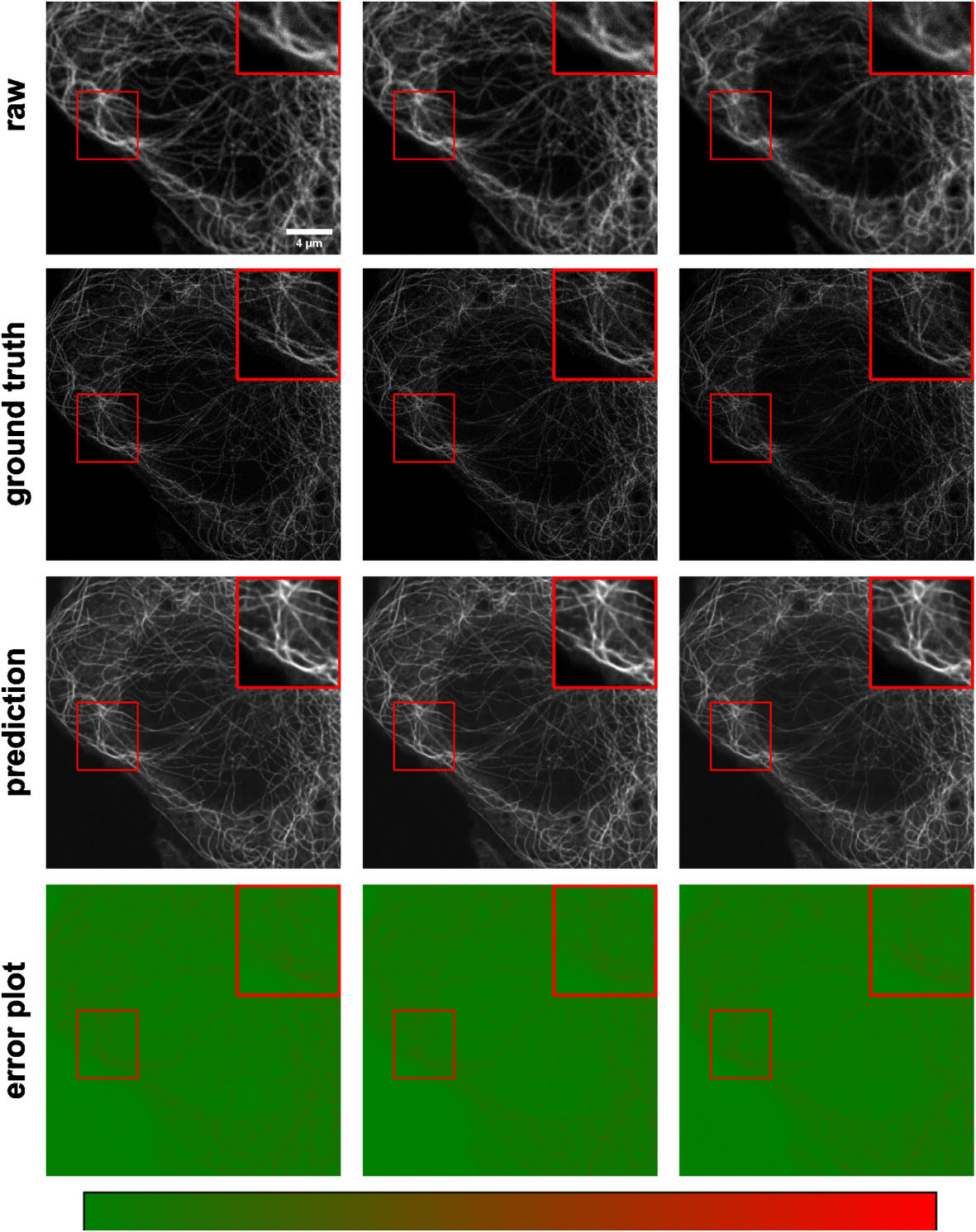
Example results of confocal-to-STED modality transformation of microtubule in 3 consecutive Z-slices. From top to bottom: raw images, reference STED images, predicted images, error plots. For the error plots, the ground-truth images were normalized as described in the main text. The contrast of grayscale images was adjusted using ImageJ’s autoscale.

**Figure 11:**
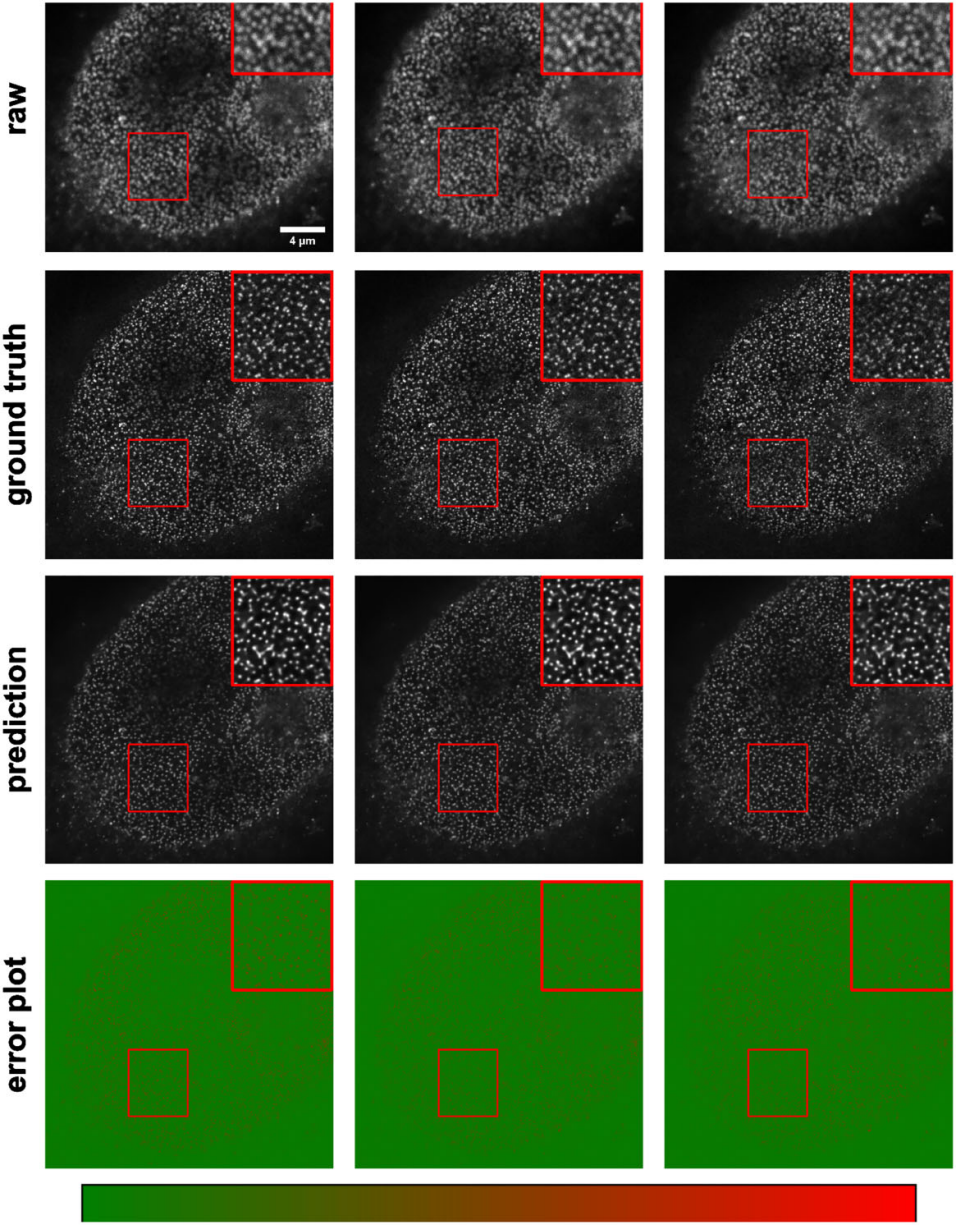
Example results of confocal-to-STED modality transformation nuclear pore in 3 consecutive Z-slices. From top to bottom: raw images, reference STED images, predicted images, error plots. For the error plots, the ground-truth images were normalized as described in the main text. The contrast of grayscale images was adjusted using ImageJ’s autoscale.

Another important application of image-to-image transformation is imaging modality transformation [8], usually from one “cheaper” modality with lower resolution (e.g., with larger FOV, easier to acquire and scale up) to another modality with higher resolution but expensive to obtain. Such models will permit a new way in assay development strategy to take advantage of all the benefits of the cheaper modality with lower resolution and still be able to enhance the resolution computationally post hoc. To demonstrate the application of *MMV_Im2Im* in this scenario, we took an example dataset with paired 3D confocal and stimulated emission depletion (STED) images of 2 different cellular structures, microtubule and nuclear pore [8]. Sample results are summarized in Figs. 10 and 11. The corresponding error plots show pixel-based absolute differences between ground truth and prediction. Intensities were normalized to the interval from −1 to 1 for training, with intensity limits restricted to the 0.01 percentile and 99.99 percentile values of the intensity distribution.

For microtubule, the model achieved Pearson correlation of 0.786 ± 0.020 and a peak signal-to-noise ratio of 21.201 ± 0.586, while for nuclear pore complex, the Pearson correlation was 0.744 ± 0.025 and the peak signal-to-noise ratio was 22.939 ± 1.896. Considering a Pearson correlation of 0.699 ± 0.030 and a peak signal-to-noise ratio of 18.847 ± 0.649 for the microtubule dataset and a Pearson correlation of 0.656 ± 0.033 and a peak signal-to-noise ratio of 20.352 ± 1.009 of the lower-resolution raw images with the higher-resolution ground truth, this approach improved data quality. Also, visual inspection can confirm the effectiveness of the models. Again, it would be necessary to conduct further quantitative evaluation to ensure the validity of users’ specific problems.

### Staining transformation in multiplex experiments

**Figure 12:**
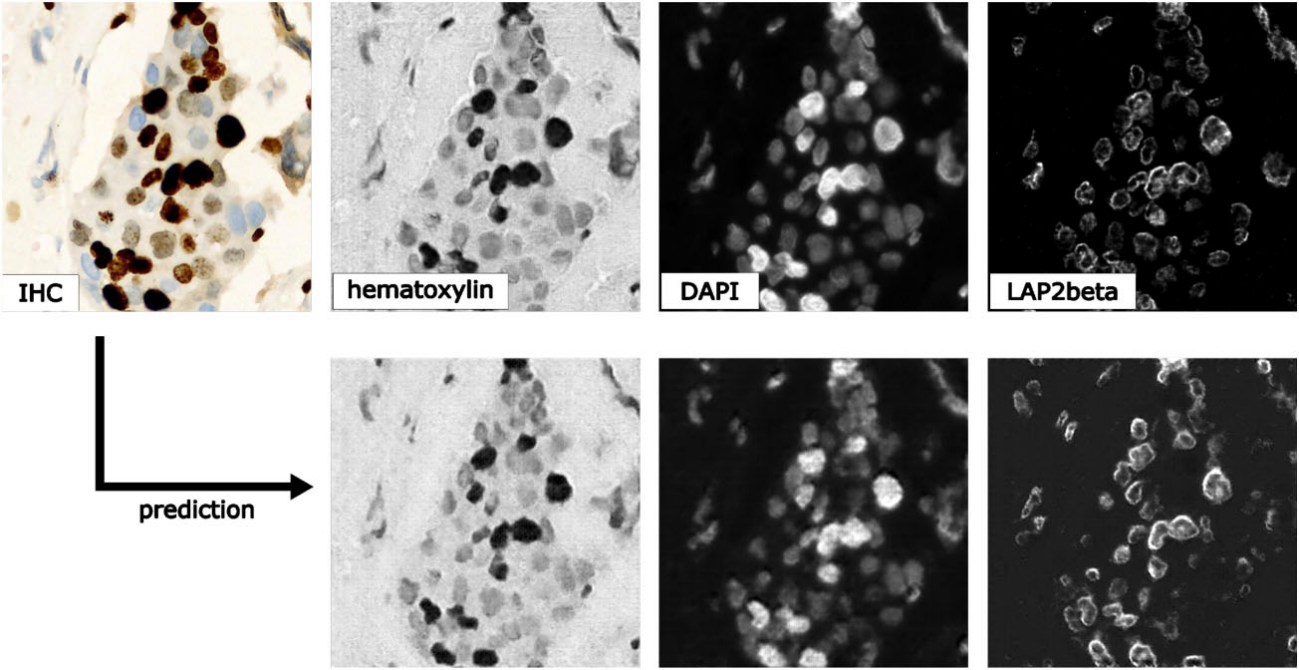
Qualitative visualization of staining transformation results with the *MMV_Im2Im* package. The top row refers to the input image (IHC) and to the respective ground truth for hematoxylin, DAPI, and LAP2beta, while the bottom row shows the respective prediction.

DL has emerged as a powerful tool for multiplex imaging, a powerful technique that enables the simultaneous detection and visualization of multiple biomolecules within a single tissue sample. This technique is increasingly being used in biomedical experiments but demands efficient image analysis solutions to accurately identify and quantify the different biomolecules of interest at scale. DL has demonstrated great potential in analyzing multiplex datasets, as it can automatically learn the complex relationships between different biomolecules and their spatial distribution within tissues. Specifically, in this study, we present the effectiveness of *MMV_Im2Im* in transforming tissue images from one staining to another, which will permit efficient coregistration, colocalization, and quantitative analysis of multiplex datasets. We used the sample dataset from [5]. In this example, we trained 3 different models to transform IHC (Immunohistochemistry) images to images of standard hematoxylin stain, mpIF nuclear (DAPI), and mpIF LAP2beta (a nuclear envelope stain). Example results can be observed in Fig. 12 to verify the results qualitatively, and the respective metrics can be found in Table 4. It is worth mentioning that there is a pixel shift in the mpIF LAP2beta holdout dataset, but image registration is beyond the scope of this article. We show the metrics as an example of an evaluation of the transformed images, but we leave an application-specific evaluation to the appropriate researchers. But it is evident that these transformed images can provide valuable insights into the localization and expression patterns of specific biomolecules spatially.

**Table 4:** Results of the staining transformation in multiplex experiments, derived from 51 holdout images each

Dataset	Pearson correlation	Structural similarity	Peak signal-to-noise ratio
Hematoxylin	0.860 ± 0.075	0.453 ± 0.063	23.855 ± 1.742
DAPI	0.920 ± 0.049	0.770 ± 0.067	26.754 ± 2.129
LAP2beta	0.435 ± 0.087	0.597 ± 0.083	22.415 ± 1.586

### Overview of used frameworks

From all experiments above (37 in total), we want to demonstrate the great flexibility of *MMV_Im2Im* and not to optimize every task in detail. Presenting all detailed configurations in these 37 experiments in the article could lead to more confusion than clarity. To this end, we give a high-level overview of the key information of each task in Table 5, hoping to serve as a valuable starting point for researchers to optimize their DL-based image-to-image transformation using *MMV_Im2Im*. The full configuration details are available in human-readable formats in our GitHub repository [22].

**Table 5:** Overview of the used frameworks for the demonstrated tasks

Task	Dimension	Framework	Backbone
Label-free	2D/3D	FCN, Pix2pix, CycleGAN	fnet, UNet, AttentionUnet, SwinUNETR, …
Semantic segmentation	2D/3D	FCN, CycleGAN	AttentionUnet, DynUnet, UNet3D
Instance segmentation	2D/3D	EmbedSeg	BranchedERFNet_2d, UNet3D
Synthetic	2D/3D	Pix2pix	AttentionUnet, fnet
Denoising	3D	FCN	UNet
Modality transformation	3D	FCN	UNet3D
Staining transformation	2D	Pix2pix	predefined_unet

## Methods

### Overview of the codebase

Overall, the package inherited the boilerplate concept from pytorch-lightning [14] and was made fully configurable via yaml files supported by pyrallis [47], as well as largely employed state-of-the-art DL components from MONAI [19]. The 3 key parts in the package, mmv_im2im.models, mmv_im2im.data_modules, and Trainers, will be further described below.

### Main frameworks for *mmv_im2im.models*

*mmv_im2im.models* is the core module defining the DL framework for your problem, where we can instantiate the neural network architecture and define what to do before training starts, what to do in each training and validation step, what to do at the end of each epoch, and so on. All are implemented following the same lightning module from pytorch-lightning, which makes the code very easy to read, to understand, and even to extend.

In general, there are mainly 4 major DL frameworks that can be applied to microscopy image-to-image transformation: supervised learning with a fully convolutional network (FCN)–type model, supervised learning with pix2pix-type models, unsupervised learning to learn mapping between visual domains, and Self2Self-type self-supervised learning [48]. The major difference between FCN-based supervised learning and pix2pix-based supervised learning is that the pix2pix framework extends an FCN model with an adversarial head as a discriminator to further improve the realism of the prediction. The major difference between the unsupervised framework and the self-supervised framework is that the unsupervised methods still require examples of the target images, even though the source images and target images do not need to be from the same sample or pixel-wise aligned. But, the self-supervised framework would only need the original images, which could be really helpful when it is impossible to acquire the target images (e.g., there is no truly noise-free or artifact-free image).

Currently, for supervised frameworks, both the FCN type and pix2pix type are well supported in the *MMV_Im2Im* (RRID:SCR_024630) package. Since our package is designed in a very generic way, it is possible to continuously expand the functionalities when available (ideally with community contributions). For example, diffusion models [49] can be thought of as a modern extension of the pix2pix-type framework and therefore are within our horizon to include into *MMV_Im2Im*. For the unsupervised framework, only CycleGAN-type methods are supported. We are planning to extend the unsupervised framework with Imaginaire [50], which will greatly extend the applicability of *MMV_Im2Im* (e.g., learning the transformation from one single image to another single image or one set of images to another set of images). Meanwhile, supporting the self-supervised framework will be our next major milestone.

### Customized *mmv_im2im.data_modules* for bioimaging applications

The *data_modules* implements a general module for data handling for all different frameworks mentioned above, from how to load the data to how to set up the dataloader for training and validation. Different people may prefer to organize their training data in different ways, such as using csv to organize input and the corresponding ground truth, or making different folders (e.g., “image” and “ground_truth”) with input and the corresponding ground truth sharing the same file name, and so on. Or some people may prefer to do a random train/validation split, while others like to presplit train and validation into different folders, and so forth. Currently, the data_module in *MMV_Im2Im* supports 4 different ways of data loading, where we try to cover as many common scenarios as possible, so that everyone will feel comfortable using it.

A big challenge in the dataloader in bioimaging applications is that there could be not only a large amount of files but files of very large sizes. To deal with each individual large image, we used the delayed loading from aicsimageio for efficient image reading. Besides, we adopted the PersistentDataloader from MONAI to further optimize the efficiency. In specific, after loading a large image and running through all the deterministic operations, like intensity normalization or spatial padding, the PersistentDataLoader will pickle and save the data in a temporary folder, to avoid repeating the heavy computation on large files in each training iteration. To handle the potentially large number of files, we implemented the data_module with the capability of loading only a certain portion of the data into the memory in each epoch and reloading with a different portion every certain number of epochs. By doing this, we were able to efficiently train an instance segmentation model with more than 125 K images, where each raw image is about 15 MB.

### State-of-the-art training with the pytorch-lightning Trainer

We fully adopted the Trainer from pytorch-lightning, which has been widely used by the machine learning community and widely tested on both R&D problems and industrial-scale applications. In a nutshell, simply by specifying the training parameters in the yaml file, users can set up multi-GPU training, half-precision training, automatic learning rate finder, automatic batch size finder, early stopping, stochastic weight averaging, and so on. This allows users to focus on the research problems without worrying about the ML engineering.

## Discussion

In this work, we presented a new open-source Python package *MMV_Im2Im* package for image-to-image transformations in bioimaging applications. We demonstrated the applicability on more than 10 different problems or datasets to give biomedical researchers a holistic view of the general image-to-image transformation concepts with diverse examples. This package was not a simple collection of existing methods. Instead, we distilled the knowledge from existing methods and created this generic version with state-of-the-art ML engineering techniques, which made the package easy to understand, easy to use, and easy to extend for future. We hope this package can serve as the starting point for other researchers doing AI-based image-to-image transformation research and eventually build a large shared community in the field of image-to-image transformation for bioimaging.

### Further works

One of main directions for extending *MMV_Im2Im* is to prepack common bioimaging datasets as a Dataset module, so that DL researchers can use it for algorithm development and benchmarking, and new users can easily use it for learning microscopy image-to-image transformation. We will continue improving the functionalities of the package, such as supporting more models and methods, such as diffusion-based models [49], unsupervised denoising [45], or Imaginaire [50]. Besides, we also plan to develop 2 auxiliary packages, *MMV_Im2Im_Auto* and *MMV_Im2Im_Active*. In specific, when you have a reasonable amount of training data, *MMV_Im2Im_Auto* will take advantage of the fact that *MMV_Im2Im* is fully configurable with yaml files and automatically generates a set of potentially good configurations, then find the optimal solution for you by cross-validation. On the other hand, when you only have very limited training data, or even with only pseudo ground truth, *MMV_Im2Im_Active* will help to build preliminary models from the limited training data and gradually refine the model with human-in-the-loop by active learning [21]. All the packages will also be wrapped into napari plugins [51] to allow no-code operation and therefore be more friendly to users without experience in programming.

The image-to-image transformation frameworks implemented in the current version do not explicitly take temporal information into account. We treat images (2D or 3D) at each time step independently. Thanks to the flexibility of aicsimageio, our package can directly read even multichannel 3D time-lapse data (i.e, 5D) during training or inference, if necessary. But the computation is done at individual time steps. A common method to integrate the temporal context with spatial information is the convolutional recurrent neural network (CRNN) [52]. The support of CRNN will be part of our future work.

Another type of microscopy image analysis problem related to image-to-image transformation is image registration, where we learn how to transform the “floating” image spatially so that it is optimally aligned with the reference image in the physical space. Recent methods are able to transform the floating image into its registered version through deep neural networks [53]. This will be another important direction for future extension.

Beyond *MMV_Im2Im*, we hope to develop a similar package for other problems (without reinventing wheels). For example, as we mentioned in the instance segmentation application, Mask-RCNN type models are also very powerful instance segmentation methods and, in theory, can also be generalized beyond 2D images. However, Mask-RCNN would fit more to a detection framework, instead of image-to-image transformation. It will be supported in our *MMV_NDet* (NDet = N-dimensional detection) package, currently under development.

## Code Availability and Requirements

- Project name: MMV_Im2Im (Microscopy Machine Vision, Image-to-Image transformation)
- Project homepage: [22]
- Operating system(s): Linux and Windows (when using GPU), also MacOS (when only using CPU)
- Programming language: Python
- Other requirements: PyTorch 2.0.1 or higher, PyTorch Lightning >2.0.0, and all other additional dependencies are specified as in [22]
- License: MIT license

To enhance the accessibility and traceability of our toolbox, we registered it with biotools (bio.tools ID: biotools:mmv_im2im) and workflow hub [54].

## Supplementary Material

giad120_GIGA-D-23-00068_Original_Submission

giad120_GIGA-D-23-00068_Revision_1

giad120_GIGA-D-23-00068_Revision_2

giad120_GIGA-D-23-00068_Revision_3

giad120_Response_to_Reviewer_Comments_Original_Submission

giad120_Response_to_Reviewer_Comments_Revision_1

giad120_Response_to_Reviewer_Comments_Revision_2

giad120_Reviewer_1_Report_Original_Submission Chris Armit -- 5/14/2023 Reviewed

giad120_Reviewer_1_Report_Revision_1 Chris Armit -- 10/10/2023 Reviewed

giad120_Reviewer_2_Report_Original_Submission Estibaliz GÃ^3^mez-de-Mariscal -- 5/29/2023 Reviewed

giad120_Reviewer_2_Report_Revision_1 Estibaliz GÃ^3^mez-de-Mariscal -- 10/9/2023 Reviewed

## Data Availability

In general, all data used in this work were from open-accessible public repositories, released with other publications under open-source licenses. All data used in this work were only for research purposes, and we confirm that we did not use these for any other noncommercial purpose or commercial purpose. The scripts we used to download and reorganize the data can be found in the release branch called “paper_version” within our repository [22]. Detailed information about each dataset is listed below, in the same order as the Results section. Snapshots of our code and other data further supporting this work are openly available in the *GigaScience* repository, GigaDB [55]. In addition, we deposited all the trained models and sample data at Zenodo [56] to ensure the reproducibility of our work. **2D:** The data were downloaded from [28] and [57]. We used all the data from the 2 sources, while 15% of the data were held out for testing. In specific, for data source 1 [28], it contains a time-lapse tiff of 240 time steps, each with 5 channels (only channels 3 and 5 were used in this work). Channel 1: Low Contrast Digital Phase Contrast (DPC) Channel 2: High Contrast DPC Channel 3: Brightfield (the input in our study) Channel 4: EGFP-α-tubulin Channel 5: mCherry-H2B (the ground truth in our study) For data source 2 [57], it contains 2 subfolders (train and test), each with snapshots sliced from different time-lapse data. Each snapshot is saved as 6 different tiff files (only the _bf and the second channel of _fluo were used in this work): _bf: bright field (the input in our study) _cyto: cytoplasm segmentation mask _dpc: phase contrast _fluo: 2 channels, first cytoplasm, second H2B (the H2B channel is the ground truth in our study) _nuclei: nuclei segmentation mask _sqrdpc: square-root phase contrast **3D:** The data were downloaded from the hiPSC single-cell image dataset from the Allen Cell Quilt Bucket [58], which was released with the publication [29]. Each FOV is a multichannel 3D image, of which the brightfield and the corresponding structure channels were used as input and ground truth, respectively. Experiments were done on 4 different cell lines: fibrillarin (structure_name = “FBL”), nucleophosmin (structure_name = “NPM1”), lamin b1 (structure_name = “LMNB1”), and histone H2B (structure_name = “HIST1H2BJ”), with 20% of the data held out for testing. These data were originally used for the MICCAI GlaS challenge [59] and are also available from a number of other sources [60, 61]. We had 1 training set (85 images) and 2 test sets (60 and 20 images). We kept the same train/test split as in the challenge. **2D:** The data were downloaded from [62] for segmenting *C. elegans* from widefield images [63]. We used all images from the dataset, while 5% of the data were held out for testing. **3D:** The data were downloaded from the hiPSC single-cell image dataset from the Allen Cell Quilt Bucket: [58]. We used the lamin b1 cell line (structure_name = “LMNB1”) for these experiments. Each raw FOV is a multichannel 3D image (DNA dye channel, membrane dye channel, structure channel, and brightfield channel), with the instance segmentation of all nuclei available. In our 2 experiments, we used the DNA dye channel and the brightfield channel as input, respectively, while using the same 3D instance segmentation ground truth. Twenty percent of the data were held out for testing. The data were downloaded from the hiPSC single-cell image dataset from the Allen Cell Quilt Bucket: [58]. We used the fibrillarin cell line (structure_name = “FBL”) for these experiments. Each raw FOV is a multichannel 3D image (DNA dye channel, membrane dye channel, structure channel, and brightfield channel). The input is always the structure channel. Then, we used the FBL_fine workflow in the Allen Cell and Structure Segmenter [21] to generate the semantic segmentation ground truth, and we used the cell instance segmentation to group fibrillarin segmentations belonging to the same cell as unique instances (see more details in the Results section), which will be used as the instance segmentation ground truth. The FBL_fine segmentation workflow was optimized for this cell line, which can be considered a good approximation of the real truth. To be conservative, we excluded images where the mean intensity of the structure channel is outside the range of [450,500], so that the results from the FBL_fine workflow can be a better approximation of the real truth. After removing the “outlier” images, we held out 20% of the data for testing. **2D:** The data were downloaded from the hiPSC single-cell image dataset from the Allen Cell Quilt Bucket: [58]. We used the tight junction cell line (structure_name = “TJP1”) for this experiment. The original image and corresponding structure segmentation were both in 3D. We took the max intensity projection of the raw structure channel and the corresponding structure segmentation for experimenting unsupervised 2D segmentation. The correspondence between images and segmentations was shuffled to simulate unpaired ground truth. Twenty percent of the data were held out for testing. **3D:** The data were also downloaded from the hiPSC single-cell image dataset from the Allen Cell Quilt Bucket: [58]. We used 3 different cell lines for these experiments: Golgi (structure_name = “ST6GAL1”), mitochondria (structure_name = “TOMM20”), and histone H2B (structure_name = “HIST12BJ”). For Golgi and mitochondria, we simply used the corresponding structure segmentation from the dataset. For histone H2B, we took the released nuclear instance segmentation and converted it to binary as semantic segmentation results. The correspondence between images and segmentations was shuffled to simulate unpaired ground truth. Twenty percent of the data were held out for testing. **2D:** The data were downloaded from the hiPSC single-cell image dataset from the Allen Cell Quilt Bucket: [58]. We used the nucleophosmin cell line (structure_name = “NPM1”) for this experiment. The original image and corresponding structure segmentation were both in 3D. We took the max intensity projection of the raw structure channel and the corresponding structure segmentation for this experiment. The input is binary segmentation, while the ground truth is the raw image. **3D:** The data were downloaded from the hiPSC single-cell image dataset from the Allen Cell Quilt Bucket: [58]. We used the histone H2B cell line (structure_name = “HIST1H2BJ”) for these experiments. For the experiment with coarse masks, we used the binarized nuclear segmentation as the input, while for the experiment with detailed masks, we used the structure segmentation of H2B as the input. The ground truth is always the raw 3D structure image. The data were downloaded from [64], which was released with the publication [46]. We used 2 datasets, “Denoising_Planaria.tar.gz” and “Denoising_Tribolium.tar.gz.” We kept the original train/test splitting in the datasets. The data were downloaded from [65], which was released with the publication [8]. We used 2 datasets, *Microtubule* and *Nuclear_Pore_complex*, from “Confocal_2_STED.zip.” We kept the original train/test splitting in the datasets. This dataset was downloaded from [66], which was released with the publication [5]. We used the dataset “BC-DeepLIIF_Training_Set.zip” and “BC-DeepLIIF_Validation_Set.zip.” In our 3 experiments, we always used the IHC image as the input and standard hematoxylin stain image, mpIF nuclear image, and mpIF LAP2beta image as ground truth, correspondingly. To help researchers get started with our tool, we have deposited all models used in the manuscript as well as sample data at [56].

## Abbreviations

AI: artificial intelligence; CRNN: convolutional recurrent neural network; DL: deep FCN: fully convolutional network; GPU: graphics processing unit; hiPSC: human induced pluripotent stem cell; ML: machine learning; mpIF: multiplex immunofluorescence; R&D: research and development; STED: stimulated emission depletion.
