# Supplementary material for: MMV_Im2Im: an open-source microscopy machine vision toolbox for image-to-image transformation
Source: Gigascience. 2024 Jan 27;13:giad120. doi: 10.1093/gigascience/giad120 (PMC10821710; doi:10.1093/gigascience/giad120)
Supplement: giad120_Response_to_Reviewer_Comments_Original_Submission [file giad120_response_to_reviewer_comments_original_submission.pdf]

Author's Response To Reviewer Comments

I have attached a PDF file for point-by-point response to reviewers' comments, as supplementary material in the submission system.
