# Supplementary material for: MMV_Im2Im: an open-source microscopy machine vision toolbox for image-to-image transformation
Source: Gigascience. 2024 Jan 27;13:giad120. doi: 10.1093/gigascience/giad120 (PMC10821710; doi:10.1093/gigascience/giad120)
Supplement: giad120_Response_to_Reviewer_Comments_Revision_1 [file giad120_response_to_reviewer_comments_revision_1.pdf]

Author's Response To Reviewer Comments

I uploaded a separated document as supplementary, which provide a point-by-point responce to reviewers' comments.
