# Supplementary material for: MMV_Im2Im: an open-source microscopy machine vision toolbox for image-to-image transformation
Source: Gigascience. 2024 Jan 27;13:giad120. doi: 10.1093/gigascience/giad120 (PMC10821710; doi:10.1093/gigascience/giad120)
Supplement: giad120_Response_to_Reviewer_Comments_Revision_2 [file giad120_response_to_reviewer_comments_revision_2.pdf]

Author's Response To Reviewer Comments

only minor changes on reference according to editor's feedback
