# Supplementary material for: MMV_Im2Im: an open-source microscopy machine vision toolbox for image-to-image transformation
Source: Gigascience. 2024 Jan 27;13:giad120. doi: 10.1093/gigascience/giad120 (PMC10821710; doi:10.1093/gigascience/giad120)
Supplement: giad120_Reviewer_1_Report_Original_Submission — Chris Armit -- 5/14/2023 Reviewed [file giad120_reviewer_1_report_original_submission.pdf]

Reviewer Report

Title: MMV_Im2Im: An Open Source Microscopy Machine Vision Toolbox for Image-to-Image Transformation

Version: Original Submission **Date:** 5/14/2023

Reviewer name: Chris Armit

Reviewer Comments to Author:

This Technical Note describes the MMV_Im2Im Open Source Microscopy Machine Vision Toolbox for Image-to-Image Transformation. The manuscript includes a variety of use case scenarios, which is impressive, and includes reuse of image data from an hiPSC single cell image dataset provided by the Allen Institute for Cell Science, and H&E image data used for the MICCAI Glas challenge. Additional use case scenarios include imaging modality transformation from 3D confocal microscopy images to stimulated emission depletion (STED) microscopy images, and denoising of image data from Planaria (flatworms) and Tribolium (beetles). The Python package for this software tool is available from GitHub (https://github.com/mmv-lab/mmv_im2im), and has been ascribed an Open Source Initiative-approved MIT license. In addition, the authors are to be commended for submitting supporting DOME-ML annotations, which provide more detail of the machine learning approach utilised by MMV_Im2Im, to the DOME-ML Registry.Major commentMy major criticism is that I was unable to install the MMV_Im2Im software tool. As part of this review, I attempted to install the software on MacOS 12.3 (Monterey) with Apple M1 chip by following the instructions on the GitHub repository.For researchers who are not computer scientists and who may wish to reuse the tool, I see great value in containerising the software tool in a Docker container. This would make installation of the software tool much more straightforward. Would the authors consider Dockerising the MMV_Im2Im software tool in an effort to encourage reuse?I detail below the readout I get when attempting to install the MMV_Im2Im software using Anaconda on MacOS 12.3 (Monterey) with Apple M1 chip. There is an error when installing the package numcodecs.**********Python 3.10.9 (main, Mar 1 2023, 12:20:14) [Clang 14.0.6]Type "copyright", "credits" or "license" for more information.IPython 8.12.0 -- An enhanced Interactive Python.pip install mmv_im2imCollecting mmv_imcimDownloading mmv_im2im-0.4.0-py2.py3-none-any.whl (75 kB)â”• 75.6/75.6 kB 1.9 MB/s eta 0:00:00[?25hCollecting protobuf<4.21.0Downloading protobuf-3.20.3-py2.py3-none-any.whl (162 kB)â”• 162.1/162.1 kB 4.1 MB/s eta 0:00:00[?25hCollecting monai>=1.1.0Downloading monai-1.1.0-202212191849-py3-none-any.whl (1.2 MB)â”• 1.2/1.2 MB 18.0 MB/s eta 0:00:00ollecting einopsDownloading einops-0.6.1-py3-none-any.whl (42 kB)â”•

”•â”•â”•â”•â”•â”•â”•â”•â”•â”•â”• 42.2/42.2 kB 1.5 MB/s eta 0:00:00[?25hCollecting scikit-imageDownloading scikit_image-0.20.0-cp310-cp310-macosx_12_0_arm64.whl (12.7 MB)â”• 12.7/12.7 MB 51.1 MB/s eta 0:00:00[?25hCollecting pyrallisDownloading pyrallis-0.3.1-py3-none-any.whl (33 kB)Collecting pytorch-lightningDownloading pytorch_lightning-2.0.2-py3-none-any.whl (719 kB)â”• 719.0/719.0 kB 21.6 MB/s eta 0:00:00[?25hCollecting numbaDownloading numba-0.57.0-cp310-cp310-macosx_11_0_arm64.whl (2.5 MB)â”• 2.5/2.5 MB 31.8 MB/s eta 0:00:00[?25hCollecting pandasDownloading pandas-2.0.1-cp310-cp310-macosx_11_0_arm64.whl (10.8 MB)â”• 10.8/10.8 MB 43.0 MB/s eta 0:00:00[?25hCollecting aicsimageioDownloading aicsimageio-4.11.0-py2.py3-none-any.whl (129 kB)â”• 129.5/129.5 kB 5.9 MB/s eta 0:00:00[?25hCollecting scikit-learnDownloading scikit_learn-1.2.2-cp310-cp310-macosx_12_0_arm64.whl (8.5 MB)â”• 8.5/8.5 MB 50.0 MB/s eta 0:00:00[?25hRequirement already satisfied: numpy>=1.17 in ./anaconda3/lib/python3.10/site-packages (from monai>=1.1.0->mmv_im2im) (1.24.3)Requirement already satisfied: torch>=1.8 in ./anaconda3/lib/python3.10/site-packages (from monai>=1.1.0->mmv_im2im) (1.13.1)Collecting ome-types>=0.3.3Downloading ome_types-0.3.4-py3-none-any.whl (142 kB)â”• 143.0/143.0 kB 6.1 MB/s eta 0:00:00irement already satisfied: wrapt>=1.12 in ./anaconda3/lib/python3.10/site-packages (from aicsimageio->mmv_im2im) (1.14.1)Collecting imagecodecs>=2020.5.30Downloading imagecodecs-2023.3.16-cp310-cp310-macosx_11_0_arm64.whl (11.5 MB)â”• 11.5/11.5 MB 3.1 MB/s eta 0:00:00irement already satisfied: lxml<5,>=4.6 in ./anaconda3/lib/python3.10/site-packages (from aicsimageio->mmv_im2im) (4.9.2)Collecting fsspec>=2022.8.0Downloading fsspec-2023.5.0-py3-none-any.whl (160 kB)â”• 160.1/160.1 kB 6.2 MB/s eta 0:00:00[?25hCollecting zarr<3,>=2.6Downloading zarr-2.14.2-py3-none-any.whl (203 kB)â”• 203.3/203.3 kB 8.3 MB/s eta 0:00:00ollecting ome-zarr>=0.6.1Downloading ome_zarr-0.7.1-py3-none-any.whl (35 kB)Collecting tifffile>=2021.8.30Downloading tifffile-2023.4.12-py3-none-any.whl (219 kB)â”• 219.4/219.4 kB 9.0 MB/s eta 0:00:00[?25hCollecting

dask[array]>=2021.4.1Downloading dask-2023.5.0-py3-none-any.whl (1.2 MB)â”• 1.2/1.2 MB 31.6 MB/s eta 0:00:00[?25hCollecting resource-backed-dask-array>=0.1.0Downloading resource_backed_dask_array-0.1.0-py2.py3-none-any.whl (8.0 kB)Collecting xarray<2023.02.0,>=0.16.1Downloading xarray-2023.1.0-py3-none-any.whl (973 kB)â”• 973.1/973.1 kB 27.3 MB/s eta 0:00:00ollecting xmlschemaDownloading xmlschema-2.2.3-py3-none-any.whl (355 kB)â”• 355.5/355.5 kB 14.1 MB/s eta 0:00:00[?25hCollecting llvmlite<0.41,>=0.40.0dev0Downloading llvmlite-0.40.0-cp310-cp310-macosx_11_0_arm64.whl (28.1 MB)â”• 28.1/28.1 MB 45.8 MB/s eta 0:00:00[?25hRequirement already satisfied: pytz>=2020.1 in ./anaconda3/lib/python3.10/site-packages (from pandas->mmv_im2im) (2022.7)Collecting tzdata>=2022.1Downloading tzdata-2023.3-py2.py3-none-any.whl (341 kB)â”• 341.8/341.8 kB 13.5 MB/s eta 0:00:00irement already satisfied: python-dateutil>=2.8.2 in ./anaconda3/lib/python3.10/site-packages (from pandas->mmv_im2im) (2.8.2)Collecting typing-inspectDownloading typing_inspect-0.8.0-py3-none-any.whl (8.7 kB)Requirement already satisfied: pyyaml in ./anaconda3/lib/python3.10/site-packages (from pyrallis->mmv_im2im) (6.0)Requirement already satisfied: tqdm>=4.57.0 in ./anaconda3/lib/python3.10/site-packages (from pytorch-lightning->mmv_im2im) (4.65.0)Requirement already satisfied: typing-extensions>=4.0.0 in ./anaconda3/lib/python3.10/site-packages (from pytorch-lightning->mmv_im2im) (4.5.0)Collecting lightning-utilities>=0.7.0Downloading lightning_utilities-0.8.0-py3-none-any.whl (20 kB)Collecting torchmetrics>=0.7.0Downloading torchmetrics-0.11.4-py3-none-any.whl (519 kB)â”• 519.2/519.2 kB 16.6 MB/s eta 0:00:00irement already satisfied: packaging>=17.1 in ./anaconda3/lib/python3.10/site-packages (from pytorch-lightning->mmv_im2im) (23.0)Collecting scipy>=1.8Downloading scipy-1.10.1-cp310-cp310-macosx_12_0_arm64.whl (28.8 MB)â”• 28.8/28.8 MB 29.9 MB/s eta 0:00:00[?25hCollecting PyWavelets>=1.1.1Downloading PyWavelets-1.4.1-cp310-cp310-macosx_11_0_arm64.whl (4.3 MB)â”• 4.3/4.3 MB 47.1 MB/s eta 0:00:00ollecting lazy_loader>=0.1Downloading lazy_loader-0.2-py3-none-any.whl (8.6 kB)Collecting networkx>=2.8Downloading networkx-3.1-py3-none-any.whl (2.1 MB)â”• 2.1/2.1 MB 36.5 MB/s eta 0:00:00[?25hCollecting imageio>=2.4.1Downloading imageio-2.28.1-py3-none-any.whl (3.4 MB)â”• 3.4/3.4 MB 44.8 MB/s eta 0:00:00irement already satisfied: pillow>=9.0.1 in ./anaconda3/lib/python3.10/site-packages (from scikit-image->mmv_im2im)

(9.4.0)Collecting joblib>=1.1.1Downloading joblib-1.2.0-py3-none-any.whl (297
kB)â”•â
”•â”•â”•â”•â”•â”•â”•â”• 298.0/298.0 kB 11.3 MB/s eta 0:00:00ollecting
threadpoolctl>=2.0.0Downloading threadpoolctl-3.1.0-py3-none-any.whl (14 kB)Requirement already
satisfied: cloudpickle>=1.5.0 in ./anaconda3/lib/python3.10/site-packages (from dask[array]>=2021.4.1-
>aicsimageio->mmv_im2im) (2.2.1)Requirement already satisfied: toolz>=0.10.0 in
./anaconda3/lib/python3.10/site-packages (from dask[array]>=2021.4.1->aicsimageio->mmv_im2im)
(0.12.0)Requirement already satisfied: click>=8.0 in ./anaconda3/lib/python3.10/site-packages (from
dask[array]>=2021.4.1->aicsimageio->mmv_im2im) (8.0.4)Requirement already satisfied: importlib-
metadata>=4.13.0 in ./anaconda3/lib/python3.10/site-packages (from dask[array]>=2021.4.1-
>aicsimageio->mmv_im2im) (6.0.0)Collecting partd>=1.2.0Downloading partd-1.4.0-py3-none-any.whl
(18 kB)Requirement already satisfied: requests in ./anaconda3/lib/python3.10/site-packages (from
fsspec>=2022.8.0->aicsimageio->mmv_im2im) (2.29.0)Collecting
aiohttp!=4.0.0a0,!=4.0.0a1Downloading aiohttp-3.8.4-cp310-cp310-macosx_11_0_arm64.whl (336
kB)â”•â
”•â”•â”•â”•â”•â”•â”•â”• 336.9/336.9 kB 13.4 MB/s eta 0:00:00[?25hCollecting
pydantic[email]<2.0,>=1.0Downloading pydantic-1.10.7-cp310-cp310-macosx_11_0_arm64.whl (2.5
MB)â”•
â”•â”•â”•â”•â”•â”•â”•â”•â”• 2.5/2.5 MB 29.4 MB/s eta 0:00:00[?25hCollecting
pint>=0.15Downloading Pint-0.21-py3-none-any.whl (286
kB)â”•â
”•â”•â”•â”•â”•â”•â”•â”• 286.2/286.2 kB 11.7 MB/s eta 0:00:00[?25hCollecting
distributedDownloading distributed-2023.5.0-py3-none-any.whl (966
kB)â”•â
”•â”•â”•â”•â”•â”•â”•â”• 966.6/966.6 kB 27.1 MB/s eta 0:00:00irement already satisfied: six>=1.5
in ./anaconda3/lib/python3.10/site-packages (from python-dateutil>=2.8.2->pandas->mmv_im2im)
(1.16.0)Collecting elementpath<5.0.0,>=4.0.0Downloading elementpath-4.1.2-py3-none-any.whl (216
kB)â”•â
”•â”•â”•â”•â”•â”•â”•â”•â”• 216.3/216.3 kB 9.4 MB/s eta 0:00:00[?25hCollecting
numcodecs>=0.10.0Downloading numcodecs-0.11.0.tar.gz (4.5
MB)â”•
â”•â”•â”•â”•â”•â”•â”•â”•â”•â”• 4.5/4.5 MB 23.6 MB/s eta 0:00:00[?25h Installing build
dependencies ... [?25ldoneetting requirements to build wheel ... [?25ldonestalling backend
dependencies ... [?25ldoneg metadata (pyproject.toml) ... [?25ldoneollecting asciitreeDownloading
asciitree-0.3.3.tar.gz (4.0 kB)Preparing metadata (setup.py) ... eollecting fastenersDownloading
fasteners-0.18-py3-none-any.whl (18 kB)Requirement already satisfied: mypy-extensions>=0.3.0 in
./anaconda3/lib/python3.10/site-packages (from typing-inspect->pyrallis->mmv_im2im)
(0.4.3)Collecting yarl<2.0,>=1.0Downloading yarl-1.9.2-cp310-cp310-macosx_11_0_arm64.whl (62
kB)â”•â
”•â”•â”•â”•â”•â”•â”•â”•â”• 62.6/62.6 kB 2.6 MB/s eta 0:00:00[?25hCollecting
multidict<7.0,>=4.5Downloading multidict-6.0.4-cp310-cp310-macosx_11_0_arm64.whl (29
kB)Requirement already satisfied: charset-normalizer<4.0,>=2.0 in ./anaconda3/lib/python3.10/site-

packages (from aiohttp!=4.0.0a0,!=4.0.0a1->fsspec>=2022.8.0->aicsimageio->mmv_im2im) (2.0.4)Collecting async-timeout<5.0,>=4.0.0a3Downloading async_timeout-4.0.2-py3-none-any.whl (5.8 kB)Requirement already satisfied: attrs>=17.3.0 in ./anaconda3/lib/python3.10/site-packages (from aiohttp!=4.0.0a0,!=4.0.0a1->fsspec>=2022.8.0->aicsimageio->mmv_im2im) (22.1.0)Collecting frozenlist>=1.1.1Downloading frozenlist-1.3.3-cp310-cp310-macosx_11_0_arm64.whl (34 kB)Collecting aiosignal>=1.1.2Downloading aiosignal-1.3.1-py3-none-any.whl (7.6 kB)Collecting s3fsDownloading s3fs-2023.5.0-py3-none-any.whl (28 kB)Requirement already satisfied: zipp>=0.5 in ./anaconda3/lib/python3.10/site-packages (from importlib-metadata>=4.13.0->dask[array]>=2021.4.1->aicsimageio->mmv_im2im) (3.11.0)Requirement already satisfied: entrypoints in ./anaconda3/lib/python3.10/site-packages (from numcodecs>=0.10.0->zarr<3,>=2.6->aicsimageio->mmv_im2im) (0.4)Collecting locketDownloading locket-1.0.0-py2.py3-none-any.whl (4.4 kB)Collecting email-validator>=1.0.3Downloading email_validator-2.0.0.post2-py3-none-any.whl (31 kB)Collecting tblib>=1.6.0Downloading tblib-1.7.0-py2.py3-none-any.whl (12 kB)Requirement already satisfied: jinja2>=2.10.3 in ./anaconda3/lib/python3.10/site-packages (from distributed->ome-zarr>=0.6.1->aicsimageio->mmv_im2im) (3.1.2)Collecting msgpack>=1.0.0Downloading msgpack-1.0.5-cp310-cp310-macosx_11_0_arm64.whl (70 kB)â”• 70.2/70.2 kB 2.9 MB/s eta 0:00:00irement already satisfied: urllib3>=1.24.3 in ./anaconda3/lib/python3.10/site-packages (from distributed->ome-zarr>=0.6.1->aicsimageio->mmv_im2im) (1.26.15)Requirement already satisfied: sortedcontainers>=2.0.5 in ./anaconda3/lib/python3.10/site-packages (from distributed->ome-zarr>=0.6.1->aicsimageio->mmv_im2im) (2.4.0)Requirement already satisfied: tornado>=6.0.3 in ./anaconda3/lib/python3.10/site-packages (from distributed->ome-zarr>=0.6.1->aicsimageio->mmv_im2im) (6.2)Requirement already satisfied: psutil>=5.7.0 in ./anaconda3/lib/python3.10/site-packages (from distributed->ome-zarr>=0.6.1->aicsimageio->mmv_im2im) (5.9.0)Collecting zict>=2.2.0Downloading zict-3.0.0-py2.py3-none-any.whl (43 kB)â”• 43.3/43.3 kB 1.6 MB/s eta 0:00:00irement already satisfied: certifi>=2017.4.17 in ./anaconda3/lib/python3.10/site-packages (from requests->fsspec>=2022.8.0->aicsimageio->mmv_im2im) (2023.5.7)Requirement already satisfied: idna<4,>=2.5 in ./anaconda3/lib/python3.10/site-packages (from requests->fsspec>=2022.8.0->aicsimageio->mmv_im2im) (3.4)Collecting dnspython>=2.0.0Downloading dnspython-2.3.0-py3-none-any.whl (283 kB)â”• 283.7/283.7 kB 11.3 MB/s eta 0:00:00[?25hRequirement already satisfied: MarkupSafe>=2.0 in ./anaconda3/lib/python3.10/site-packages (from jinja2>=2.10.3->distributed->ome-zarr>=0.6.1->aicsimageio->mmv_im2im) (2.1.1)Collecting aiobotocore~=2.5.0Downloading aiobotocore-2.5.0-py3-none-any.whl (72 kB)â”• 72.7/72.7 kB 3.0 MB/s eta 0:00:00[?25hCollecting botocore<1.29.77,>=1.29.76Downloading botocore-1.29.76-py3-none-any.whl (10.4 MB)â”• 10.4/10.4 MB 42.0 MB/s eta 0:00:00[?25hCollecting

aioitertools>=0.5.1Downloading aioitertools-0.11.0-py3-none-any.whl (23 kB)Collecting jmespath<2.0.0,>=0.7.1Downloading jmespath-1.0.1-py3-none-any.whl (20 kB)Building wheels for collected packages: numcodecs, asciitreeBuilding wheel for numcodecs (pyproject.toml) ... [?25lerrorerror: subprocess-exited-with-errorÃ— Building wheel for numcodecs (pyproject.toml) did not run successfully.â”‚ exit code: 1â•°â”€> [186 lines of output]/private/var/folders/2b/06vzqlbs7v79yn23c9ngxcw80000gn/T/pip-build-env-vguqygr9/overlay/lib/python3.10/site-packages/setuptools/config/pyprojecttoml.py:66: _BetaConfiguration: Support for `[tool.setuptools]` in `pyproject.toml` is still *beta*.config = read_configuration(filepath, True, ignore_option_errors, dist)[numcodecs] setting up Blosc extension[numcodecs] compiling Blosc extension without SSE2 support[numcodecs] compiling Blosc extension without AVX2 support[numcodecs] setting up Zstandard extension[numcodecs] setting up LZ4 extension[numcodecs] setting up compat extension[numcodecs] setting up shuffle extension[numcodecs] setting up vlen extensionrunning bdist_wheelrunning buildrunning build_pycreating buildcreating build/lib.macosx-11.1-arm64-cpython-310creating build/lib.macosx-11.1-arm64-cpython-310/numcodecscopying ./numcodecs/lzma.py -> build/lib.macosx-11.1-arm64-cpython-310/numcodecscopying ./numcodecs/astype.py -> build/lib.macosx-11.1-arm64-cpython-310/numcodecscopying ./numcodecs/gzip.py -> build/lib.macosx-11.1-arm64-cpython-310/numcodecscopying ./numcodecs/base64.py -> build/lib.macosx-11.1-arm64-cpython-310/numcodecscopying ./numcodecs/bz2.py -> build/lib.macosx-11.1-arm64-cpython-310/numcodecscopying ./numcodecs/version.py -> build/lib.macosx-11.1-arm64-cpython-310/numcodecscopying ./numcodecs/compat.py -> build/lib.macosx-11.1-arm64-cpython-310/numcodecscopying ./numcodecs/checksum32.py -> build/lib.macosx-11.1-arm64-cpython-310/numcodecscopying ./numcodecs/ndarray_like.py -> build/lib.macosx-11.1-arm64-cpython-310/numcodecscopying ./numcodecs/quantize.py -> build/lib.macosx-11.1-arm64-cpython-310/numcodecscopying ./numcodecs/registry.py -> build/lib.macosx-11.1-arm64-cpython-310/numcodecscopying ./numcodecs/pickles.py -> build/lib.macosx-11.1-arm64-cpython-310/numcodecscopying ./numcodecs/fixedscaleoffset.py -> build/lib.macosx-11.1-arm64-cpython-310/numcodecscopying ./numcodecs/__init__.py -> build/lib.macosx-11.1-arm64-cpython-310/numcodecscopying ./numcodecs/bitround.py -> build/lib.macosx-11.1-arm64-cpython-310/numcodecscopying ./numcodecs/zlib.py -> build/lib.macosx-11.1-arm64-cpython-310/numcodecscopying ./numcodecs/shuffle.py -> build/lib.macosx-11.1-arm64-cpython-310/numcodecscopying ./numcodecs/delta.py -> build/lib.macosx-11.1-arm64-cpython-310/numcodecscopying ./numcodecs/zfpy.py -> build/lib.macosx-11.1-arm64-cpython-310/numcodecscopying ./numcodecs/msgpacks.py -> build/lib.macosx-11.1-arm64-cpython-310/numcodecscopying ./numcodecs/packbits.py -> build/lib.macosx-11.1-arm64-cpython-310/numcodecscopying ./numcodecs/categorize.py -> build/lib.macosx-11.1-arm64-cpython-310/numcodecscopying ./numcodecs/json.py -> build/lib.macosx-11.1-arm64-cpython-310/numcodecscopying ./numcodecs/abc.py -> build/lib.macosx-11.1-arm64-cpython-310/numcodecscreating build/lib.macosx-11.1-arm64-cpython-310/numcodecs/testscopying ./numcodecs/tests/test_categorize.py -> build/lib.macosx-11.1-arm64-cpython-310/numcodecs/testscopying ./numcodecs/tests/test_registry.py -> build/lib.macosx-11.1-arm64-cpython-310/numcodecs/testscopying ./numcodecs/tests/test_vlen_utf8.py -> build/lib.macosx-11.1-

arm64-cpython-310/numcodecs/testscopying ./numcodecs/tests/test_bitround.py -> build/lib.macosx-11.1-arm64-cpython-310/numcodecs/testscopying ./numcodecs/tests/test_gzip.py -> build/lib.macosx-11.1-arm64-cpython-310/numcodecs/testscopying ./numcodecs/tests/test_delta.py -> build/lib.macosx-11.1-arm64-cpython-310/numcodecs/testscopying ./numcodecs/tests/test_lzma.py -> build/lib.macosx-11.1-arm64-cpython-310/numcodecs/testscopying ./numcodecs/tests/test_bz2.py -> build/lib.macosx-11.1-arm64-cpython-310/numcodecs/testscopying ./numcodecs/tests/__init__.py -> build/lib.macosx-11.1-arm64-cpython-310/numcodecs/testscopying ./numcodecs/tests/test_pickles.py -> build/lib.macosx-11.1-arm64-cpython-310/numcodecs/testscopying ./numcodecs/tests/test_quantize.py -> build/lib.macosx-11.1-arm64-cpython-310/numcodecs/testscopying ./numcodecs/tests/test_lz4.py -> build/lib.macosx-11.1-arm64-cpython-310/numcodecs/testscopying ./numcodecs/tests/test_msgpacks.py -> build/lib.macosx-11.1-arm64-cpython-310/numcodecs/testscopying ./numcodecs/tests/test_astype.py -> build/lib.macosx-11.1-arm64-cpython-310/numcodecs/testscopying ./numcodecs/tests/test_packbits.py -> build/lib.macosx-11.1-arm64-cpython-310/numcodecs/testscopying ./numcodecs/tests/common.py -> build/lib.macosx-11.1-arm64-cpython-310/numcodecs/testscopying ./numcodecs/tests/test_base64.py -> build/lib.macosx-11.1-arm64-cpython-310/numcodecs/testscopying ./numcodecs/tests/test_shuffle.py -> build/lib.macosx-11.1-arm64-cpython-310/numcodecs/testscopying ./numcodecs/tests/test_fixedscaleoffset.py -> build/lib.macosx-11.1-arm64-cpython-310/numcodecs/testscopying ./numcodecs/tests/test_compat.py -> build/lib.macosx-11.1-arm64-cpython-310/numcodecs/testscopying ./numcodecs/tests/test_zlib.py -> build/lib.macosx-11.1-arm64-cpython-310/numcodecs/testscopying ./numcodecs/tests/test_json.py -> build/lib.macosx-11.1-arm64-cpython-310/numcodecs/testscopying ./numcodecs/tests/test_ndarray_like.py -> build/lib.macosx-11.1-arm64-cpython-310/numcodecs/testscopying ./numcodecs/tests/test_vlen_array.py -> build/lib.macosx-11.1-arm64-cpython-310/numcodecs/testscopying ./numcodecs/tests/test_zstd.py -> build/lib.macosx-11.1-arm64-cpython-310/numcodecs/testscopying ./numcodecs/tests/test_vlen_bytes.py -> build/lib.macosx-11.1-arm64-cpython-310/numcodecs/testscopying ./numcodecs/tests/test_entrypoints.py -> build/lib.macosx-11.1-arm64-cpython-310/numcodecs/testscopying ./numcodecs/tests/test_checksum32.py -> build/lib.macosx-11.1-arm64-cpython-310/numcodecs/testscopying ./numcodecs/tests/test_zfpy.py -> build/lib.macosx-11.1-arm64-cpython-310/numcodecs/testscopying ./numcodecs/tests/test_blosc.py -> build/lib.macosx-11.1-arm64-cpython-310/numcodecs/testsrunning egg_infowriting numcodecs.egg-info/PKG-INFO/private/var/folders/2b/06vzqlbs7v79yn23c9ngxcw80000gn/T/pip-build-env-vguqygr9/overlay/lib/python3.10/site-packages/setuptools/dist.py:183: SetuptoolsDeprecationWarning: Invalid config.!!**
newlines are not allowed in `summary` and will break in the future
!!write_field('Summary', single_line(summary))writing dependency_links to numcodecs.egg-info/dependency_links.txtwriting requirements to numcodecs.egg-info/requires.txtwriting top-level names to numcodecs.egg-info/top_level.txtlisting git files failed - pretending there aren't anyreading manifest file 'numcodecs.egg-info/SOURCES.txt'reading manifest template 'MANIFEST.in'adding license file 'LICENSE.txt'writing manifest file 'numcodecs.egg-info/SOURCES.txt'running build_extbuilding

'numcodecs.blosc' extensioncreating build/temp.macosx-11.1-arm64-cpython-310creating build/temp.macosx-11.1-arm64-cpython-310/c-blosccreating build/temp.macosx-11.1-arm64-cpython-310/c-blosc/blosccreating build/temp.macosx-11.1-arm64-cpython-310/c-blosc/internal-complibscreating build/temp.macosx-11.1-arm64-cpython-310/c-blosc/internal-complibs/lz4-1.9.3creating build/temp.macosx-11.1-arm64-cpython-310/c-blosc/internal-complibs/zlib-1.2.8creating build/temp.macosx-11.1-arm64-cpython-310/c-blosc/internal-complibs/zstd-1.4.8creating build/temp.macosx-11.1-arm64-cpython-310/c-blosc/internal-complibs/zstd-1.4.8/commoncreating build/temp.macosx-11.1-arm64-cpython-310/c-blosc/internal-complibs/zstd-1.4.8/compresscreating build/temp.macosx-11.1-arm64-cpython-310/c-blosc/internal-complibs/zstd-1.4.8/decompresscreating build/temp.macosx-11.1-arm64-cpython-310/c-blosc/internal-complibs/zstd-1.4.8/dictBuildercreating build/temp.macosx-11.1-arm64-cpython-310/numcodecsclang -Wno-unused-result -Wsign-compare -Wunreachable-code -DNDEBUG -fwrapv -O2 -Wall -fPIC -O2 -isystem /Users/chrisarmit/anaconda3/include -arch arm64 -fPIC -O2 -isystem /Users/chrisarmit/anaconda3/include -arch arm64 -DHAVE_LZ4=1 -DHAVE_ZLIB=1 -DHAVE_ZSTD=1 -Ic-blosc/blosc -Ic-blosc/internal-complibs/lz4-1.9.3 -Ic-blosc/internal-complibs/zstd-1.4.8 -Ic-blosc/internal-complibs/zlib-1.2.8 -Ic-blosc/internal-complibs/zstd-1.4.8/compress -Ic-blosc/internal-complibs/zstd-1.4.8/dictBuilder -Ic-blosc/internal-complibs/zstd-1.4.8/decompress -Ic-blosc/internal-complibs/zstd-1.4.8/legacy -Ic-blosc/internal-complibs/zstd-1.4.8/common -Ic-blosc/internal-complibs/zstd-1.4.8/dll -Ic-blosc/internal-complibs/zstd-1.4.8/deprecated -Ic-blosc/internal-complibs/zstd-1.4.8/dll/example -I/Users/chrisarmit/anaconda3/include/python3.10 -c c-blosc/blosc/bitshuffle-generic.c -o build/temp.macosx-11.1-arm64-cpython-310/c-blosc/blosc/bitshuffle-generic.o -stdlib=libc++xcode-select: note: no developer tools were found at '/Applications/Xcode.app', requesting install. Choose an option in the dialog to download the command line developer tools.[numcodecs] command '/usr/bin/clang' failed with exit code 1Traceback (most recent call last):File "/private/var/folders/2b/06vzqlbs7v79yn23c9ngxcw80000gn/T/pip-build-env-vguqygr9/overlay/lib/python3.10/site-packages/setuptools/_distutils/unixccompiler.py", line 185, in _compileself.spawn(compiler_so + cc_args + [src, '-o', obj] + extra_postargs)File "/private/var/folders/2b/06vzqlbs7v79yn23c9ngxcw80000gn/T/pip-build-env-vguqygr9/overlay/lib/python3.10/site-packages/setuptools/_distutils/ccompiler.py", line 1041, in spawnspawn(cmd, dry_run=self.dry_run, **kwargs)File "/private/var/folders/2b/06vzqlbs7v79yn23c9ngxcw80000gn/T/pip-build-env-vguqygr9/overlay/lib/python3.10/site-packages/setuptools/_distutils/spawn.py", line 70, in spawnraise DistutilsExecError(distutils.errors.DistutilsExecError: command '/usr/bin/clang' failed with exit code 1During handling of the above exception, another exception occurred:Traceback (most recent call last):File "<string>", line 258, in build_extensionFile "/private/var/folders/2b/06vzqlbs7v79yn23c9ngxcw80000gn/T/pip-build-env-vguqygr9/overlay/lib/python3.10/site-packages/setuptools/command/build_ext.py", line 246, in build_extension_build_ext.build_extension(self, ext)File "/private/var/folders/2b/06vzqlbs7v79yn23c9ngxcw80000gn/T/pip-build-env-vguqygr9/overlay/lib/python3.10/site-packages/setuptools/_distutils/command/build_ext.py", line 548, in build_extensionobjects = self.compiler.compile(File "/private/var/folders/2b/06vzqlbs7v79yn23c9ngxcw80000gn/T/pip-build-env-

vguqygr9/overlay/lib/python3.10/site-packages/setuptools/_distutils/ccompiler.py", line 600, in
compileself._compile(obj, src, ext, cc_args, extra_postargs, pp_opts)File
"/private/var/folders/2b/06vzqlbs7v79yn23c9ngxcw80000gn/T/pip-build-env-
vguqygr9/overlay/lib/python3.10/site-packages/setuptools/_distutils/unixccompiler.py", line 187, in
_compileraise CompileError(msg)distutils.errors.CompileError: command '/usr/bin/clang' failed with exit
code 1During handling of the above exception, another exception occurred:Traceback (most recent call
last):File "/Users/chrisarmit/anaconda3/lib/python3.10/site-
packages/pip/_vendor/pep517/in_process/_in_process.py", line 351, in <module>main()File
"/Users/chrisarmit/anaconda3/lib/python3.10/site-
packages/pip/_vendor/pep517/in_process/_in_process.py", line 333, in mainjson_out['return_val'] =
hook(**hook_input['kwargs'])File "/Users/chrisarmit/anaconda3/lib/python3.10/site-
packages/pip/_vendor/pep517/in_process/_in_process.py", line 249, in build_wheelreturn
_build_backend().build_wheel(wheel_directory, config_settings,File
"/private/var/folders/2b/06vzqlbs7v79yn23c9ngxcw80000gn/T/pip-build-env-
vguqygr9/overlay/lib/python3.10/site-packages/setuptools/build_meta.py", line 416, in
build_wheelreturn self._build_with_temp_dir(['bdist_wheel'], '.whl',File
"/private/var/folders/2b/06vzqlbs7v79yn23c9ngxcw80000gn/T/pip-build-env-
vguqygr9/overlay/lib/python3.10/site-packages/setuptools/build_meta.py", line 401, in
_build_with_temp_dirself.run_setup()File
"/private/var/folders/2b/06vzqlbs7v79yn23c9ngxcw80000gn/T/pip-build-env-
vguqygr9/overlay/lib/python3.10/site-packages/setuptools/build_meta.py", line 338, in
run_setupexec(code, locals())File "<string>", line 284, in <module>File "<string>", line 275, in
run_setupFile "/private/var/folders/2b/06vzqlbs7v79yn23c9ngxcw80000gn/T/pip-build-env-
vguqygr9/overlay/lib/python3.10/site-packages/setuptools/__init__.py", line 107, in setupreturn
distutils.core.setup(**attrs)File "/private/var/folders/2b/06vzqlbs7v79yn23c9ngxcw80000gn/T/pip-
build-env-vguqygr9/overlay/lib/python3.10/site-packages/setuptools/_distutils/core.py", line 185, in
setupreturn run_commands(dist)File
"/private/var/folders/2b/06vzqlbs7v79yn23c9ngxcw80000gn/T/pip-build-env-
vguqygr9/overlay/lib/python3.10/site-packages/setuptools/_distutils/core.py", line 201, in
run_commandsdist.run_commands()File
"/private/var/folders/2b/06vzqlbs7v79yn23c9ngxcw80000gn/T/pip-build-env-
vguqygr9/overlay/lib/python3.10/site-packages/setuptools/_distutils/dist.py", line 969, in
run_commandsself.run_command(cmd)File
"/private/var/folders/2b/06vzqlbs7v79yn23c9ngxcw80000gn/T/pip-build-env-
vguqygr9/overlay/lib/python3.10/site-packages/setuptools/dist.py", line 1244, in
run_commandsuper().run_command(command)File
"/private/var/folders/2b/06vzqlbs7v79yn23c9ngxcw80000gn/T/pip-build-env-
vguqygr9/overlay/lib/python3.10/site-packages/setuptools/_distutils/dist.py", line 988, in
run_commandcmd_obj.run()File "/private/var/folders/2b/06vzqlbs7v79yn23c9ngxcw80000gn/T/pip-
build-env-vguqygr9/normal/lib/python3.10/site-packages/wheel/bdist_wheel.py", line 343, in
runself.run_command("build")File "/private/var/folders/2b/06vzqlbs7v79yn23c9ngxcw80000gn/T/pip-
build-env-vguqygr9/overlay/lib/python3.10/site-packages/setuptools/_distutils/cmd.py", line 318, in

run_commandself.distribution.run_command(command)File
"/private/var/folders/2b/06vzqlbs7v79yn23c9ngxcw80000gn/T/pip-build-env-
vguqygr9/overlay/lib/python3.10/site-packages/setuptools/dist.py", line 1244, in
run_commandsuper().run_command(command)File
"/private/var/folders/2b/06vzqlbs7v79yn23c9ngxcw80000gn/T/pip-build-env-
vguqygr9/overlay/lib/python3.10/site-packages/setuptools/_distutils/dist.py", line 988, in
run_commandcmd_obj.run()File "/private/var/folders/2b/06vzqlbs7v79yn23c9ngxcw80000gn/T/pip-
build-env-vguqygr9/overlay/lib/python3.10/site-packages/setuptools/_distutils/command/build.py",
line 131, in runself.run_command(cmd_name)File
"/private/var/folders/2b/06vzqlbs7v79yn23c9ngxcw80000gn/T/pip-build-env-
vguqygr9/overlay/lib/python3.10/site-packages/setuptools/_distutils/cmd.py", line 318, in
run_commandself.distribution.run_command(command)File
"/private/var/folders/2b/06vzqlbs7v79yn23c9ngxcw80000gn/T/pip-build-env-
vguqygr9/overlay/lib/python3.10/site-packages/setuptools/dist.py", line 1244, in
run_commandsuper().run_command(command)File
"/private/var/folders/2b/06vzqlbs7v79yn23c9ngxcw80000gn/T/pip-build-env-
vguqygr9/overlay/lib/python3.10/site-packages/setuptools/_distutils/dist.py", line 988, in
run_commandcmd_obj.run()File "<string>", line 251, in runFile
"/private/var/folders/2b/06vzqlbs7v79yn23c9ngxcw80000gn/T/pip-build-env-
vguqygr9/overlay/lib/python3.10/site-packages/setuptools/command/build_ext.py", line 84, in
run_build_ext.run(self)File "/private/var/folders/2b/06vzqlbs7v79yn23c9ngxcw80000gn/T/pip-build-
env-vguqygr9/overlay/lib/python3.10/site-packages/setuptools/_distutils/command/build_ext.py", line
345, in runself.build_extensions()File
"/private/var/folders/2b/06vzqlbs7v79yn23c9ngxcw80000gn/T/pip-build-env-
vguqygr9/overlay/lib/python3.10/site-packages/setuptools/_distutils/command/build_ext.py", line 467,
in build_extensionsself._build_extensions_serial()File
"/private/var/folders/2b/06vzqlbs7v79yn23c9ngxcw80000gn/T/pip-build-env-
vguqygr9/overlay/lib/python3.10/site-packages/setuptools/_distutils/command/build_ext.py", line 493,
in _build_extensions_serialself.build_extension(ext)File "<string>", line 261, in
build_extension__main__.BuildFailed[end of output]note: This error originates from a subprocess, and
is likely not a problem with pip.ERROR: Failed building wheel for numcodecsuilding wheel for asciitree
(setup.py) ... [?25ldonereated wheel for asciitree: filename=asciitree-0.3.3-py3-none-any.whl size=5032
sha256=053f680f0d67e28efcff6e27e7e4bc35cecb79d3970e0325974e3a5017db313fStored in directory:
/Users/chrisarmit/Library/Caches/pip/wheels/da/b9/03/c77bc170956f3789cd19dcd311e208bf6340fdd
b0a94ac6ce4Successfully built asciitreeFailed to build numcodecsERROR: Could not build wheels for
numcodecs, which is required to install pyproject.toml-based projectsNote: you may need to restart the
kernel to use updated packages.

Level of Interest

Please indicate how interesting you found the manuscript: Choose an item.

Quality of Written English

Please indicate the quality of language in the manuscript: Choose an item.

Declaration of Competing Interests

Please complete a declaration of competing interests, considering the following questions:

- Have you in the past five years received reimbursements, fees, funding, or salary from an organisation that may in any way gain or lose financially from the publication of this manuscript, either now or in the future?
- Do you hold any stocks or shares in an organisation that may in any way gain or lose financially from the publication of this manuscript, either now or in the future?
- Do you hold or are you currently applying for any patents relating to the content of the manuscript?
- Have you received reimbursements, fees, funding, or salary from an organization that holds or has applied for patents relating to the content of the manuscript?
- Do you have any other financial competing interests?
- Do you have any non-financial competing interests in relation to this paper?

If you can answer no to all of the above, write 'I declare that I have no competing interests' below. If your reply is yes to any, please give details below.

I work for GigaScience where I perform checks on the quality of image data and the image analysis approach. My funding is not dependent on the outcome of this review, and I declare that I have no competing interests.

I agree to the open peer review policy of the journal. I understand that my name will be included on my report to the authors and, if the manuscript is accepted for publication, my named report including any attachments I upload will be posted on the website along with the authors' responses. I agree for my report to be made available under an Open Access Creative Commons CC-BY license (http://creativecommons.org/licenses/by/4.0/). I understand that any comments which I do not wish to be included in my named report can be included as confidential comments to the editors, which will not be published.

Choose an item.

To further support our reviewers, we have joined with Publons, where you can gain additional credit to further highlight your hard work (see: https://publons.com/journal/530/gigascience). On publication of this paper, your review will be automatically added to Publons, you can then choose whether or not to claim your Publons credit. I understand this statement.

Yes Choose an item.
