# Supplementary material for: MMV_Im2Im: an open-source microscopy machine vision toolbox for image-to-image transformation
Source: Gigascience. 2024 Jan 27;13:giad120. doi: 10.1093/gigascience/giad120 (PMC10821710; doi:10.1093/gigascience/giad120)
Supplement: giad120_Reviewer_2_Report_Original_Submission — Estibaliz GÃ3mez-de-Mariscal -- 5/29/2023 Reviewed [file giad120_reviewer_2_report_original_submission.pdf]

Reviewer Report

Title: MMV_Im2Im: An Open Source Microscopy Machine Vision Toolbox for Image-to-Image Transformation

Version: Original Submission **Date:** 5/29/2023

Reviewer name: Estibaliz GÃ³mez-de-Mariscal

Reviewer Comments to Author:

Sonneck and Chen proposed a generic Python package (MMV_Im2Im) to solve image-to-image bioimaging tasks using machine learning techniques. MMV_Im2Im provides flexibility with machine learning engineering strategies for different image-to-image tasks. It is inspired by the OpenMMLab project (https://openmmlab.com/) and is based on libraries like PyTorch, PyTorch-Lightning and MONAI. The authors describe 10 different workflows to showcase the usability of MMV_Im2Im in six 2D and six 3D publicly available datasets.The work of Sonneck and Chen is an important contribution to speeding up future image analysis developments that aim to combine or integrate machine learning in loop. Frameworks such as MMV_Im2Im are very much needed in the community to enable more multidisciplinary biomedical imaging applications. Moreover, MMV-im2im is a rich central library for image-to-image transformations equipped with the latest models (e.g., transformers) and with the flexibility to deal with 5D data, a very much needed functionality in bioimage analysis that is not such common in natural domain image analysis. Thus, the following comments are made with the aim of improving and refine the content of the paper.**Strengths of the paper**- MMV_Im2Im is open-source, fully documented, enriched with examples and use cases. They provide parameter configuration files for both training and inference of the models, which facilitates the reproducibility of any deployed workflow.- The proposed use cases are well documented and equipped with accessorial Jupyter notebooks to reproduce the workflows.- MMV_Im2Im gathers state-of-the art solutions for different image processing tasks.**Weaknesses or major comments***Functionalities of MMV_Im2Im:*- The package is meant to analyse 2D-5D data. Assuming that 5D data comes from (3D + channels + time), it should be highlighted that none of the use-cases show an example with temporal information or where the time information is taken into consideration. Is MM2-Im2Im taking into consideration time-lapse information during normalization, data augmentation and image restoration for example? These are quite important specially to ensure consistency in the posterior quantification of the videos.- MMV_Im2Im integrates many different approaches such as EmbedSeg or Pix2Pix. This means that the package contains a large number of dependencies that in many cases may not be necessary. For instance, if a user wants to use image restoration, they wouldn't need to install EmbedSeg or MONAI. On the other hand, some of these approaches cannot be transferable to different tasks (i.e., EmbedSeg is only for segmentation). With time, all these may carry dependency conflicts. Thus, did the authors consider creating a vanilla version of the package and a lighter one? Or even more modular, a way to install MMV-Im2Im specific to the image processing task?- Related to the previous point, it is difficult to get the full potential of the library and which CNN or ML approaches are available for each image processing task. One way could be having a table that describes the type of approaches or networks that

are available.In the discussion, paragraph 2, the authors mention that MMV_Im2Im can generate a set of potentially good configurations, would not this be similar to an AutoML set up? Did the authors consider programming something similar?*Content of the manuscript:*- In page 16, paragraph "For 2D demonstration, ….", the description of the strategy to train an unsupervised approach is not clear. Please specify which network (cycleGAN?) was used in the unsupervised training.- In page 5, the authors describe the parameter configuration file. Is this configuration file also exported when training a model from the code directly? This could reinforce the future reproducibility of the training.- None of the trained models used for the use cases are provided so the results are hardly reproducible. I will recommend having all the weights in a repository and enabling their access.- In general, for all the described use cases it is difficult to know which ML pipeline was used. A way to easily provide this could be a table with the name of the proposed task, the dataset name, if it is 2D or 3D, the name of the CNN, type of normalization, and if there was any data augmentation, for example.- Page 11, in line 3, it is said "have achieved huge success in 2D semantic segmentation". I would remove "2D" as in general ML has demonstrate great potential for all types of semantic segmentations.- For the use case in Figure 5, which are the approaches used for the segmentation? The same for the example of unsupervised semantic segmentation of intracellular structures. The models and pre/post-processing routines are not specified.- For the last proposal of the use case in Figure 7, the authors provide a binary mask that also contains some textures inside the cell nuclei. How were these masks obtained? Also, this type of image transformation is meant to augment training data. That is, it can be used to virtually generate pairs of image + mask. However, only providing the fine-masks, one would still need to annotate the nuclei instances. Thus, what is the proposal in this case? How could one generate fine masks?*Related with the figures in the PDF:*- In many cases, the qualitative results cannot be correctly compared because of the scale and quality of the Figure. I would recommend using a zoom, similar to the one used in Figure 9.- Please include a scale bar in the images.- In some Figures, such as Figure 8, it is recommendable to change the Look Up Table (LUT) of the microscopy images or their contrast (by mentioning it in the Figure caption) to improve the visualization.- In Figure 6 and 9, the predicted image is similar to the ground truth. A way to improve the visualization and highlight the differences between both could be to display a color overlap, an error map or a profile plot of some specific areas in the field of view.- All the figures are divided into labelled sections (A, B, C, …), however in some cases the corresponding explanation in the caption is missing. Also, some of the images in the Figures (e.g., Figure 9) lack a corresponding label indicating the approach, network or whether the image is the result of a prediction, or it is the ground truth.- Figure 3: Was staining normalization applied to the images shown in C) (i.e. the test images)? Please, include this information in the Figure caption.- Pages 9-10: The reference to Figure 2-C (in Page 9, Line11, Word 9), should be to Figure 2-D. Then Figure 2-C would never be mentioned, does it make sense to still have it? Also, what is the content of Figure 2-D?- Page 15, Figure 6: The green and purple arrows in the image are never explained or mentioned.- Page 10, Figure 2: Yellow arrows in image A are not explained in the text nor the figure's description.- Page 17, Figure 7-A: It should be explained what each image represents.*Evaluation metrics & quantitative results:*- We agree with the authors that detailed evaluation is out of the scope of this work. However, for scientific robustness, it is always recommended to provide an intuition or some examples of how the approaches could be evaluated.- For the consistency of the report, it is recommendable to include to some extent a quantitative assessment of the results obtained. For example, there is a lack of quantitative results in

tasks (1) 2D label free, (2.3) comparison of semantic segmentation and instance segmentation, (5) generation of synthetic microscopy images and (8) stain transformation. However, the authors comment on the performance of the method by visual inspection. Such comparison can be reinforced by the computation of one dedicated measurement.- Likewise, we recommend specifying at least the standard deviation of the reported accuracy values. For example, in '2D semantic segmentation of tissues from H&E images', only the average F1-score is given, hidden further information about the model stability.- '...have you ever had the moment when you wanted to extend someone's open-source code to suit your special ML needs, but found it so difficult to figure out where and how to extend, especially for complex methods-' - Please specify that such a connection can only be made in environments using PyTorch (i.e., for TensorFlow based environments, users would need to look for other resources).Regarding GitHub's webpage (https://github.com/MMV-Lab/mmv_im2im):- The installation files and methods on the GitHub pages do not work. Because many of the library versions are not specified (pytorch_lightning, numba, scikit-image and numpy), the installation gives dependencies errors and manual fixing is required. A potential solution could be to provide the corresponding environment specification (yaml file for conda, mamba or python environment) so it is possible to reproduce locally the working environment.- The library as it is with the PyTorch configuration does not allow it's use without a GPU available, which in some cases may be an impediment for debugging or testing part of the code.*Maintenance of the proposed framework:*- DL-based image processing is a quickly evolving field, thus the versatility and success of the current framework relies on its capacity to become adapted to new DL approaches (e.g., diffusion models) and Python packages. Did the authors define any strategy or structure in the framework to be able to consume future updates from PyTorch Lighting, which is expected to bring these new approaches? If not, could the authors provide some sort of discussion about this?**Typos**- Page 4, "Bottleneck: existing codE not easy…"- Page 4, I would change "applications" by tasks in the sentence "demonstrate its applications in over 10 biomedical APPLICATIONS of various types."- Page 5, "different state-of-the-art ML TRAINING engineering techniques,…"- Page 5, Line 11 (not considering the title), Word 8: Change 'tubable' to 'tunable'.- Page 7, Figure 1: In Application 3, the image on the right (the prediction) does not match with the one on the left (the input).- Page 9, Line 3, Word 4: Change 'apprearnce' to 'appearance'.- Page 12, Line 8 "improvment" ïƒ "improvement".- Page 12, Line 20, Words 14: Change 'amound' to 'amount'.- Page 12, paragraph 4, line 7: "But, the cells touch" ïƒ "But, the cells touching ".- Page 13, Line 3, Words 6: Change ''-B' to 'Figure '-B'.- Page 14, Line 7 (not considering the title), Words 3-4: Change 'segmentation segmentation' to 'semantic segmentation'.- Page 15: In the title (Generating synthetic microscopy images from binary Masks), change 'Masks' to 'masks'.- Page 16, Line 22, Word 5: Change 'mask' to 'masks'.- Page 20, Line 3 (not considering the title), Word 8: Change 'expereiements' to 'experiments'.- Page 20, Line 5 (not considering the title), Word 8: Change 'muliplex' to 'multiplex'.- Page 21, Line 21, Word 14: Change 'an' to 'a'.- Page 22, Line 7(not considering the title), Word 10: Change 'python' to 'Python'.- Page 22, Line 28 (not considering the title), Word 11: Change 'Napari' to 'napari'.**Minor comments **- If the goal of MM2_Im2Im is that 'bioimaging researchers can really focus on research and develop novel methods for their biomedical applications, without worrying about the ML engineering works', I would recommend creating training and inference Jupyter notebooks for Google Colab in addition to all the given configurations, as many of biomedical researchers do not have a GPU in their computer. This would create a user-friendly interface, allowing additional features such as

interactive creation of configuration files.- MM2_Im2Im is meant to provide solutions for almost any Image to Image task, however, image registration is not included in the package. Although this potentially imply a significant work, I would recommend the authors to consider the integration of this task in a near future.- The current library is already exporting a configuration YAML file, which should be easily transferable to the format of the BioImage Model Zoo (https://bioimage.io/). Did the authors consider absorbing its model format? If so, users of MMV-Im2Im could benefit of having already some models accessible for fine-tuning.

Level of Interest

Please indicate how interesting you found the manuscript: Choose an item.

Quality of Written English

Please indicate the quality of language in the manuscript: Choose an item.

Declaration of Competing Interests

Please complete a declaration of competing interests, considering the following questions:

- Have you in the past five years received reimbursements, fees, funding, or salary from an organisation that may in any way gain or lose financially from the publication of this manuscript, either now or in the future?
- Do you hold any stocks or shares in an organisation that may in any way gain or lose financially from the publication of this manuscript, either now or in the future?
- Do you hold or are you currently applying for any patents relating to the content of the manuscript?
- Have you received reimbursements, fees, funding, or salary from an organization that holds or has applied for patents relating to the content of the manuscript?
- Do you have any other financial competing interests?
- Do you have any non-financial competing interests in relation to this paper?

If you can answer no to all of the above, write 'I declare that I have no competing interests' below. If your reply is yes to any, please give details below.

I declare that I have no competing interests

I agree to the open peer review policy of the journal. I understand that my name will be included on my report to the authors and, if the manuscript is accepted for publication, my named report including any attachments I upload will be posted on the website along with the authors' responses. I agree for my report to be made available under an Open Access Creative Commons CC-BY license (http://creativecommons.org/licenses/by/4.0/). I understand that any comments which I do not wish to

be included in my named report can be included as confidential comments to the editors, which will not be published.

Choose an item.

To further support our reviewers, we have joined with Publons, where you can gain additional credit to further highlight your hard work (see: https://publons.com/journal/530/gigascience). On publication of this paper, your review will be automatically added to Publons, you can then choose whether or not to claim your Publons credit. I understand this statement.

Yes Choose an item.
