# Supplementary material for: MMV_Im2Im: an open-source microscopy machine vision toolbox for image-to-image transformation
Source: Gigascience. 2024 Jan 27;13:giad120. doi: 10.1093/gigascience/giad120 (PMC10821710; doi:10.1093/gigascience/giad120)
Supplement: giad120_Reviewer_2_Report_Revision_1 — Estibaliz GÃ3mez-de-Mariscal -- 10/9/2023 Reviewed [file giad120_reviewer_2_report_revision_1.pdf]

Reviewer Report

Title: MMV_Im2Im: An Open Source Microscopy Machine Vision Toolbox for Image-to-Image Transformation

Version: Revision 1 **Date:** 10/9/2023

Reviewer name: Estibaliz GÃ³mez-de-Mariscal

Reviewer Comments to Author:

We thank the authors for the work carried out to improve the software tool, its documentation and the content of the manuscript. All our comments, both the minor and major ones, have been carefully addressed.We tested the new version of the toolbox and there are few minor technical comments that we hope the authors could fix:** Some of the tutorials and examples provided withing the GitHub repository need further curation **1. Example provided in the GitHub repository: The installation in Mac (M1) works but when running the example the following changes needed to be done:a) Model checkpoint: it does not work as no model is provided at the moment.b) After trying it with one of the models provided by the authors in this review, it is still failing, as the code is setup for a GPU connection:>>> executor.setup_model()...raise RuntimeError('Attempting to deserialize object on a CUDA 'RuntimeError: Attempting to deserialize object on a CUDA device but torch.cuda.is_available() is False. If you are running on a CPU-only machine, please use torch.load with map_location=torch.device('cpu') to map your storages to the CPU.2. On the GitHub README.md, it is said: "The installation tags, embedseg, advance, paper, all, are be selected based on your needs." However, embededseg is not an option.3. Trying to use 'run_im2im --config /paper_configs/labelfree_2d_FCN_train.yaml', outputs the following error:my_func = getattr(my_module, info["func_name"])AttributeError: module 'monai.transforms' has no attribute 'AddChanneld'This happens when installing MONAI from the dev branch of their GitHub repository, which is the one by default. It seems that in the commit https://github.com/Project-MONAI/MONAI/pull/7019 they removed the 'AddChanneld' functionality. We highly recommend the authors to ensure the installation with frozen versions of the dependencies, including MONAI.4. Some notebooks that are downloading data lack some code to create a folder or further arguments and make the notebooks to fail.5. The tutorial on https://github.com/MMV-Lab/mmv_im2im/blob/main/tutorials/quick_start.md does not work. Error because an argument on line (https://github.com/MMV-Lab/mmv_im2im/blob/9a806d0a0698bcd0f5a53b1eee35b0837e5a5c28/mmv_im2im/configs/preset_train_labelfree_3d.yaml#L76):TypeError: __init__() got an unexpected keyword argument 'gpus'5. On Docker building (on run_container.sh) for both cuda and amd64, it fails. It needs to change Â´mmv_im2im:$1Â´ on line 8 with 'mmvlab/mmv_im2im:$1'.**Paper**In the 'Instance segmentation' section, when giving the precision values, no standard deviation has been added.Despite this little changes in the GitHub repository needed for the easy and ensured usability of the tool, we consider the manuscript has addressed successfully all our concerns.IvÃ¡n Hidalgo-Cenalmor and Estibaliz GÃ³mez-de-Mariscal

Level of Interest

Please indicate how interesting you found the manuscript: Choose an item.

Quality of Written English

Please indicate the quality of language in the manuscript: Choose an item.

Declaration of Competing Interests

Please complete a declaration of competing interests, considering the following questions:

- Have you in the past five years received reimbursements, fees, funding, or salary from an organisation that may in any way gain or lose financially from the publication of this manuscript, either now or in the future?
- Do you hold any stocks or shares in an organisation that may in any way gain or lose financially from the publication of this manuscript, either now or in the future?
- Do you hold or are you currently applying for any patents relating to the content of the manuscript?
- Have you received reimbursements, fees, funding, or salary from an organization that holds or has applied for patents relating to the content of the manuscript?
- Do you have any other financial competing interests?
- Do you have any non-financial competing interests in relation to this paper?

If you can answer no to all of the above, write 'I declare that I have no competing interests' below. If your reply is yes to any, please give details below.

I declare that I have no competing interests

I agree to the open peer review policy of the journal. I understand that my name will be included on my report to the authors and, if the manuscript is accepted for publication, my named report including any attachments I upload will be posted on the website along with the authors' responses. I agree for my report to be made available under an Open Access Creative Commons CC-BY license (http://creativecommons.org/licenses/by/4.0/). I understand that any comments which I do not wish to be included in my named report can be included as confidential comments to the editors, which will not be published.

Choose an item.

To further support our reviewers, we have joined with Publons, where you can gain additional credit to further highlight your hard work (see: https://publons.com/journal/530/gigascience). On publication of

this paper, your review will be automatically added to Publons, you can then choose whether or not to claim your Publons credit. I understand this statement.

Yes Choose an item.
